# A systematic review and meta-analysis of data on pregnant women with confirmed COVID-19: Clinical presentation, and pregnancy and perinatal outcomes based on COVID-19 severity

**DOI:** 10.7189/jogh.11.05018

**Published:** 2021-06-30

**Authors:** Zohra S Lassi, Anna Ali, Jai K Das, Rehana A Salam, Zahra A Padhani, Omar Irfan, Zulfiqar A Bhutta

**Affiliations:** 1 Robinson Research Institute, the University of Adelaide, Adelaide, Australia; 2 Adelaide Medical School, the University of Adelaide, Adelaide, Australia; 3 Division of Women and Child Health, the Aga Khan University, Karachi, Pakistan; 4 Centre for Global Child Health, the Hospital for Sick Children (SickKids), Toronto, Canada; 5 Centre of Excellence in Women and Child Health, the Aga Khan University, Karachi, Pakistan

## Abstract

**Background:**

We determined the clinical presentation, risk factors, and pregnancy and perinatal outcomes in pregnant women with confirmed COVID-19 and identified if these are different based on COVID-19 severity.

**Methods:**

We included all observational studies on pregnant women with confirmed COVID-19 reporting clinical presentation, risk factors, and pregnancy and perinatal outcomes. We included all studies published between Dec/2019-Feb/2021 in Medline, Embase, the WHO COVID-19 databases, and clinicaltrials.gov. The methodological quality of cohort and case-series was assessed using NHLBI criteria.

**Results:**

31 016 pregnant women from 62 studies were included. Women were an average of 30.9 years of age, most (77.7%) were in the third trimester, and 16.4% developed severe COVID-19. Nearly half were asymptomatic, while the most commonly reported symptoms were cough, fever, fatigue, and anosmia/ageusia. About 7% were admitted to the intensive care unit (ICU), 8% required mechanical ventilation, and 2% of the women died. Almost 80% of women delivered; 48.4% had cesarean births. Among newborns, 23.4% were preterm (<37 weeks), 16.6% were low birth weight, and 23.7% were admitted to neonatal ICU. A total of 21 stillbirths (1.6%) and 24 neonatal deaths (1.6%) were recorded, while 50 babies (3.5%) were COVID-19 positive. Studies comparing pregnant women with severe and non-severe COVID-19 showed that women with severe COVID-19 were 3.7 years older and the risk of severe COVID-19 was 1.5 times higher among women >35 years. The risk of severe COVID-19 was significantly higher among women who were obese, had smoked, diabetic, and had pre-eclampsia. The risk of preterm birth was almost 2.4 folds among women with severe COVID-19.

**Conclusions:**

Our review suggests a heightened risk of COVID-19 severity and adverse pregnancy and perinatal outcomes among women with certain demographic and health profiles. These findings can inform the formation of current guidelines; however, these should be constantly updated as the global COVID-19 scenario unfolds.

**Registration:**

PROSPERO: CRD42020182048.

Since its emergence in December 2019, the novel coronavirus-2 has fast become one of the greatest global health challenges of the last century. The virus has so far (as of June 21, 2021) been spread to 216 countries and territories, infected around 167 million people, and resulted in 3.86 million deaths [1]. Because of the nature of spread, the number of cases has been exponentially increasing every day. Although the virus is prevalent in all ages regardless of gender, it is more severe and can cause deaths among those who have a pre-existing disease [2], and those who are disadvantaged and lack access and affordability to health services [3]. Majority of the deaths reported worldwide are among the elderly or those with co-morbidities and poor immunity [4]. Women during pregnancy often face several pregnancy-related complications and are more susceptible to respiratory pathogens that may place them at higher risk of adverse outcomes [5]. The new coronavirus is genetically closer to Severe Acute Respiratory Syndrome-Corona virus-1 (SARS-CoV-1), which in earlier studies have shown the high occurrence of adverse maternal and neonatal outcomes including preterm birth, intrauterine growth restriction (IUGR), endotracheal intubation, and intensive care unit (ICU) admission, with minimal chance of vertical transmission [6,7]. A recent systematic review on pregnancies infected with recent and past coronavirus-related illnesses (including SARS-CoV-1 and Middle East Respiratory Syndrome – MERS) has established a high risk of miscarriage, pre-eclampsia, preterm birth, and perinatal death [8]. However, they included very few cases of SARS-CoV-2 in their analysis (41 cases only) [8]. A more recently published review on confirmed and suspected pregnant women with COVID-19 reported that advanced maternal age, pre-existing comorbidities and increased body mass index (BMI) are the risk factors for severe COVID-19 [9].

After the emergence of COVID-19, many evidence syntheses have been published, however, the majority of those have presented limited data from case reports and case series only which lacked methodological robustness [10-13]. We found few systematic reviews with a larger population of pregnant women [9,14]. Of these reviews, one has presented the clinical and laboratory features of more than 2500 pregnant women and reported increased maternal ICU admissions among women in higher age brackets and those with comorbidities [14]. Another recent systematic review of 77 studies included women of reproductive age (both confirmed and suspected cases) and reported outcomes on pregnant and non-pregnant women [9]. Although systematic reviews on the topic exist, but with a rapid rate of accumulating evidence from larger prospective studies, it is important to frequently update the evidence to better understand the clinical presentation, risk factors, and pregnancy and perinatal outcomes in pregnant women with laboratory-confirmed COVID-19 irrespective of clinical signs and symptoms and severity of COVID-19, and at the same time, identify women at a higher risk for COVID-19 severity and its impact on pregnancy, birth, and perinatal outcomes.

## METHODS

### Eligibility criteria, study selection, information sources, search strategy

The protocol of the review has been registered with PROSPERO - CRD42020182048. A systematic review was carried out using Preferred Reporting Items for Systematic Review and Meta-analysis (PRISMA) guidelines to assess the impact of COVID-19 on pregnancy, birth, and perinatal outcomes [15]. This systematic review included large cohort and case series including multi-country and multi-center studies, describing the clinical presentation, risk factors, and pregnancy and perinatal outcomes in pregnant/postpartum women with laboratory-confirmed COVID-19 (serology or RT-PCR) irrespective of clinical signs and symptoms and severity of COVID-19 [16].

The search strategy was prepared using the MeSH and free text terms for “coronavirus” and “pregnant women” (Table S1 in the **Online Supplementary Document**) and applied to databases including Medline, EMBASE, the World Health Organization (WHO) COVID-19 global literature on coronavirus disease, China Knowledge Resource Integrated Database (CNKI), and Google Scholar. Pre-print databases MedRxiv (https://www.medrxiv.org), and BioRxiv (https://www.biorxiv.org, and ongoing registered studies on Clinicaltrials.gov were also searched. We also contacted established groups that were conducting and compiling surveillance data from large cohorts. The bibliographies of all included studies and existing systematic reviews were also hand-searched. No language restriction was applied and the review included all eligible studies published since December 2019. The last search date was Feb 15, 2021. The review excluded studies on non-pregnant women, infants (beyond neonatal age), children, and other adult populations presenting to hospitals with COVID-19 like symptoms. The review also excluded systematic and narrative reviews, guidance/guidelines, opinion papers, and commentaries with no mention of original cases. We excluded case reports identified in the searches (Table S2 in the **Online Supplementary Document**), also the studies that only reported universal screening and positive cases without any mention of clinical presentation, risk factors, and pregnancy and perinatal outcomes. For studies with overlapping cases, we contacted the authors to confirm the center name and time period in which the participants were included and judged the overlaps with other studies. When we couldn’t hear from study authors we only included the study with a larger sample size when the hospital/medical centers and the time period in which cases were included were similar, and when it wasn’t very clear we did not include the study (Table S3 in the **Online Supplementary Document**). When there was uncertainty, the corresponding author of the primary study was contacted to confirm the information.

### Data extraction and quality assessment

All identified studies were uploaded into Covidence Systematic Review Software for screening [17] and then independently sifted for relevance by two review authors (ZSL and AA) and discrepancies were resolved by discussion. Data were independently extracted from eligible studies by two authors (ZSL and AA) using the pre-designed extraction sheet. Information was extracted on publication year; location; study design; sample size; clinical presentation of COVID-19 like symptoms; past medical history (comorbidities); COVID-19 diagnosis, severity and management; pregnancy, birth, and perinatal outcomes; and neonatal COVID-19 status. The methodological quality of included studies was assessed using criteria outlined by the National Heart, Lung, and Blood Institute (NHLBI) [18].

### Data synthesis

The pooled analysis was performed using the Comprehensive Meta-Analysis (CMA) Software [19] and Joanna Briggs Institute (JBI) SUMARI software [20]. The meta-analysis pooled overall prevalence using Dersimonian and Laird random-effect meta-analysis after transforming data using Freeman-Tukey transformation arcsine square root transformation. The review pooled overall means and proportion for clinical manifestations, risk factors, pregnancy, and perinatal outcomes and reported their 95% confidence intervals (CI) and the percentage of variation across studies that is due to heterogeneity rather than chance using I^2^ statistics.

The review assessed the impact of COVID-19 severity on pregnancy, birth, and perinatal outcomes from non-comparative dichotomous data using bivariate analysis [21]. Since severity was defined differently in the included studies, therefore, for this review, we categorized COVID-19 case severity based on the WHO definition as [22]:

- Non-severe (asymptomatic/mild, and moderate cases): women with no symptoms, mild symptoms (case definition for COVID-19 without evidence of viral pneumonia or hypoxemia), and moderate symptoms (clinical signs of pneumonia with no hypoxemia on room air).
- Severe (severe and critical cases): women with dyspnoea with a respiratory rate of ≥30 breaths per minute and hypoxemia on room air, requiring oxygen therapy or mechanical (invasive or non-invasive) ventilation including extracorporeal membrane oxygenation (ECMO).

Studies that compared pregnant women with non-severe and severe COVID-19 and reported outcomes were separately pooled. The random-effects models were prepared using Review Manager 5.4 (The Nordic Cochrane Centre, The Cochrane Collaboration, Copenhagen, Denmark) [23] and the outcomes were summarized as risk ratios (RR) and mean difference (MD) with 95% CIs.

## RESULTS

### Study selection

**Figure 1:**
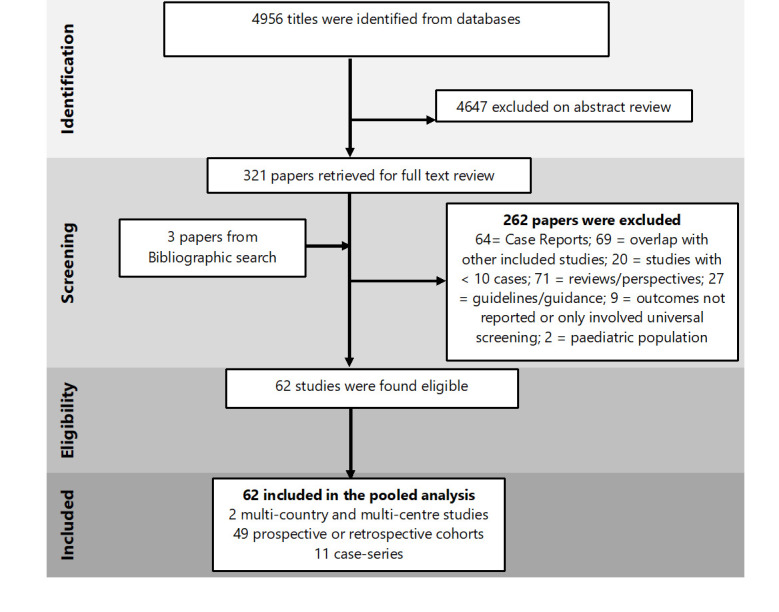
PRISMA flow diagram of study selection process.

A total of 4956 studies were identified using the search strategy, after removing the duplicates. Full texts of 321 papers were reviewed and finally, 62 studies (including 31 016 pregnant women) met the eligibility criteria (**Figure 1**).

### Characteristics of included studies

Of the 62 included studies, 11 were case-series, and 51were prospective/retrospective cohorts including two multi-country studies. Of all the studies, one paper was published in Chinese [24]. Characteristics of studies included are summarized in Table S4 in the **Online Supplementary Document**. We identified a study that has reported only on the clinical presentation of 23 434 pregnant women from the USA without mention of birth and pregnancy outcomes. Since this large USA study did overlap with other studies from the USA (included in this systematic review), we only considered this large study for calculating the pooled prevalence of the clinical presentation in pregnant women. However, for birth and perinatal outcomes, other studies from the USA were considered for pooled prevalence calculation.

**Figure 2:**
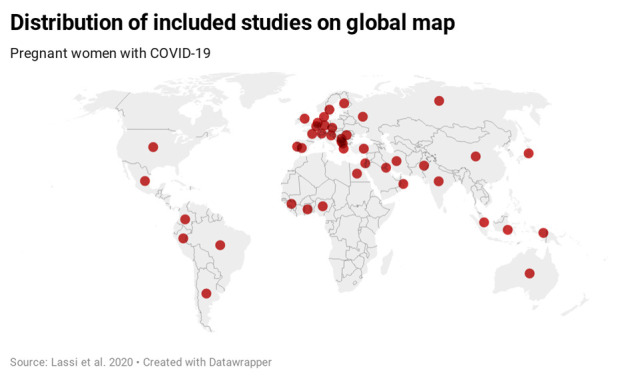
Distribution of included studies on global map.

Studies included were from 44 countries of the six continents: Australia, Africa (Egypt, French Guinea, Ghana, Nigeria), Asia (China, India, Indonesia, Iran, Japan, Kuwait, Oman, Pakistan, Russia, Singapore), Europe (Belgium, Czech Republic, Denmark, Finland, France, Germany, Greece, Israel, Italy, Moscow, North Macedonia, Portugal, Republic of Kosovo, Romania, Serbia, Slovenia, Spain, Sweden, Switzerland, the Netherlands, Turkey, the UK), North America (Dominica Republic, Mexico, the USA), and South America (Argentina, Brazil, Colombia, Peru) (**Figure 2**).

Of the 31 016 pregnant women with COVID-19, information on severity was reported for 8.8% (n = 2743; 13 studies) of the included women. Of these, 85.6% had non-severe COVID-19, and the remaining 14.4% had severe COVID-19. We performed a separate pooled analysis of 13 studies that compared pregnant women with non-severe and severe COVID-19 in the same study [24-39].

### The methodological quality of included studies

The methodological quality of included studies is assessed and reported in Tables S5a and S5b in the **Online Supplementary Document**. Almost 80% of the studies reported the research objective and population, however eligibility, blinding of assessment, and loss to follow-up were not clearly stated in the studies. Of all the case-series, the majority failed to report the statistical methods, however, the rest of the domains were reported in those studies.

### Synthesis of the results

*Sociodemographic and basic health profile of all included women:* **Table 1** presents the socio-demographic and basic health profile of pregnant women infected with COVID-19. The mean age of pregnant women presenting to the hospital was 30.92 years; of these, nearly one-third (31.3%) were older than 35 years. Their mean gestational age on presentation was 33.36 weeks, with the majority (77.7%) in the third trimester at the time of reporting. Half of all women were multiparous (51.6%), and one-third were nulliparous (34.7%). The majority of the women were Asian (61.8%), followed by Central/Latin American (58.5%), White (35.2%), and Black (15.8%). More than half of all women had public/state health insurance (53.4%) and the other half had private health insurance (49.4%). Their mean body mass index (BMI) was 27.77; of these 38.2% were overweight and 19.3% were obese or morbidly obese.

**Table 1:** Socio-demographic and health profile of pregnant women with COVID-19 – all cases

Variables		All cases (n = 31 016)			
**# studies**	**Total events/participants**	**Pooled proportion (%)**	**Heterogeneity (*I^2%^*)**
**Age (years)**	Mean age (years)	40		30.92 ± 0.65	99.7
	<35 years	12	20 476/25 256	54.9 (38.6 to 70.7)	99.0
	≥35 yearsa	10	4546/24 883	31.3 (23.5 to 39.6)	95.0
**Gestational age (Weeks)**	Mean gestational age	22		33.36 ± 0.71	99.6
	First trimester	9	184/1847	13.9 (6.4 to 23.3)	94.5
	Second trimester	11	369/1887	20.7 (13.2 to 29.2)	90.6
	Third trimester	15	1373/2184	77.7 (63.4 to 89.5)	97.5
	≥37 weeks	4	360/1120	39.3 (9.3 to 74.5)	99.0
	<37 weeks	3	602/1056	65.1 (22.9 to 96.9)	99.3
**Parity**	Nulliparous	18	667/1821	34.7 (28.4 to 41.3)	87.4
	Multiparous	9	464/887	51.6 (36.8 to 66.2)	94.7
**Ethnicity**	White	8	5919/24 962	35.2 (16.9 to 55.9)	99.3
	Black	6	3586/25 199	15.8 (7.4 to 26.5)	98.5
	Asian	6	1246/24 987	61.8 (19.3 to 95.8)	99.9
	Central/Latin American	4	7230/23 760	58.5 (28.1 to 85.7)	98.8
	Mixed	2	62/488	10.6 (8.0 to 13.6)	98.4
	Others	4	972/24 706	2.1 (0.4 to 5.0)	93.8
**Health insurance**	Private	4	196/436	49.4 (39.0 to 59.9)	75.1
	Public	4	265/476	53.4 (44.2 to 62.5)	71.9
**Body Mass Index**	Mean BMI	16		27.77 ± 0.72	99.0
	<25	2	130/435	28.7 (24.2 to 33.3)	32.4
	25 to <30	3	480/1166	38.2 (28.6 to 48.3)	89.2
	≥30	13	735/26 170	19.3 (10.5 to 30.0)	98.8

**Figure 3:**
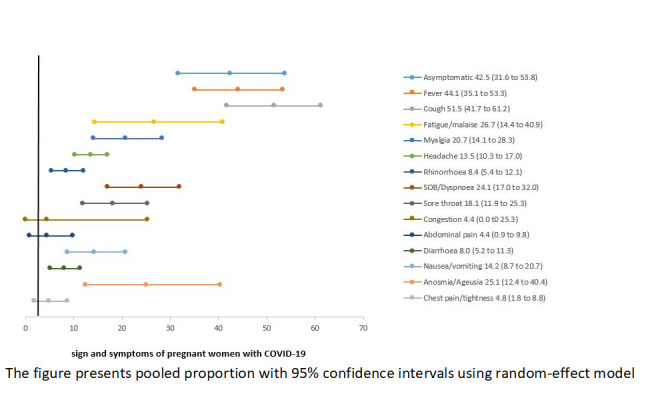
Clinical presentations on pregnant women with COVID-19. The figure presents pooled proportion with 95% confidence intervals using random-effect model.

*Clinical presentation, comorbidities, and pregnancy complications:* **Table 2** presents the clinical manifestations, past medical history, and pregnancy-related complications among pregnant women with COVID-19. While almost half (42.5%) of all women were asymptomatic, the most commonly reported symptoms were cough (51.5%), fever (44.1%), fatigue/malaise (26.7%), and anosmia/ageusia (25.1%). The other commonly reported symptoms included shortness of breath (SOB)/dyspnoea (24.1%), myalgia (20.7%), sore throat (18.1%), and nausea/vomiting (14.2%) (**Figure 3**). The risk of developing severe COVID-19 was higher among women who presented with fever, cough, fatigue/malaise, myalgia, headache, SOB/dyspnoea, diarrhea, and chest pain/tightness compared to non-severe cases. **Table 2****.**

**Table 2:** Clinical presentation, past medical history, among pregnant women with confirmed COVID-19 – all cases (n = 31 016)

Characteristics of all pregnant women		All cases (n = 31016)				Based on COVID-19 severity (n = 2743)		
**Total # of studies**	**Total events/participants**	Pooled proportion* (%) (95% CI)	**Heterogeneity (*I^2%^*)**	**Severe COVID-19**§ **(n = 395)**	**Non-severe COVID-19**‖ **(n = 2348)**	**Risk ratio (95% CI)**
Presenting symptoms	Asymptomatic	26	1274/3422	42.5 (31.6 to 53.8)	97.4	0	103/390 [27,34,36,40-42]	Non-estimable
	Fever	27	1905/26 947	44.1 (35.1 to 53.3)	98.1	44/56 [27,28,31,36,43]	239/393[27,28,31,36,38,41]	**1.29 (1.10 to 1.51)**
	Cough	26	7074/26 931	51.5 (41.7 to 61.2)	98.4	49/56 [24,27,28,36,43]	230/396[24,27,28,36,38,41]	**1.51 (1.32 to 1.72)**
	Fatigue/malaise	20	1931/25 587	26.7 (14.4 to 40.9)	98.9	20/41 [27,28,36,38]	101/313[27,28,36,38]	**1.51 (1.06 to 2.15)**
	Myalgia	12	4046/25 262	20.7 (14.1 to 28.3)	96.0	11/18 [38]	8/122 [27,38]	**9.32 (4.34 to 20.02)**
	Headache	12	4653/25 054	13.5 (10.3 to 17.0)	80.6	9/27 [36,38]	8/135 [36,38,44]	**5.63 (2.39 to 13.27)**
	Rhinorrhoea	7	1471/24 954	8.4 (5.4 to 12.1)	89.2	-	-	Non-estimable
	SOB/Dyspnoea	26	3456/26 887	24.1 (17.0 to 32.0)	97.9	38/56 [27,28,31,36,38,43]	99/393 [27,28,31,36,38,41]	**1.39 (1.17 to 1.65)**
	Sore throat	13	3195/25 188	18.1 (11.9 to 25.3)	95.8	2/8 [27]	46/68 [41]	0.37 (0.11 to 1.24)
	Congestion	2	176/23 463	4.4 (0.0 to 25.3)	91.2	-	-	Non-estimable
	Abdominal pain	4	879/23 632	4.4 (0.9 to 9.8)	79.4	-	-	-
	Nausea/vomiting	11	2231/25 010	14.2 (8.7 to 20.7)	95.1	6/6 [28]	74/128 [28,41]	Non-estimable
	Diarrhoea	20	1718/26 615	8.0 (5.2 to 11.3)	93.3	7/15 [28,36]	31/299 [27,28,36]	**4.50 (2.38 to 8.50)**
	Anosmia/ageusia	16	2712/25 637	25.1 (12.4 to 40.4)	99.1	5/6 [28]	77/97 [24,28]	1.05 (0.72 to 1.52)
	Chest pain/tightness	8	436/24 899	4.8 (1.8 to 8.8)	93.7	8/27 [36,38]	16/123 [36,38]	**2.28 (1.09 to 4.77)**
Past medical history	No comorbidity	5	172/358	40.6 (15.3 to 68.6)	94.2	-	-	-
	Smoke/tobacco	18	235/3492	8.3 (2.3 to 17.8)	98.3	14/148 [26,28,33,37]	20/683 [28,33,34,37]	**3.23 (1.67 to 6.25)**
	Anaemia	5	189/1171	16.1 (4.9 to 32.0)	96.8	3/8 [27]	0	Non-estimable
	Hypothyroidism	7	126/1270	8.7 (4.3 to 14.3)	86.0	2/14 [26,27]	7/181 [26,45]	3.69 (0.85-16.14)
	Chronic resp. disease (incl. asthma)	17	786/27 486	7.0 (2.3 to 13.5)	98.8	32/216 [26,33,35,46]	58/835 [26,33,34,38,40,44,45]	**2.13 (1.42-3.20)**
	Cardiac disease (incl. HTN)	16	462/26 750	4.4 (2.9 to 6.2)	87.1	40/293 [26,27,33,35,46,47]	43/796 [33,35,45]	**2.53 (1.68-3.81)**
	Chronic kidney disease	3	25/24 176	0.5 (0.0 to 2.5)	92.0	-	-	-
	Diabetes	18	606/27 110	3.4 (1.8 to 5.5(	92.2	7/128 [33,47]	20/777 [26,33,37,40,44,45]	2.12 (0.92 to 4.92)
	Immunocompromised	3	158/23 669	2.7 (0.0 to 12.7)	96.2	-	-	** to**
	Any chronic disease†‡	11	8413/26 240	18.9 (11.7 to 27.3)	98.2	61/212 [28,33,43,46]	61/683 [28,33,34,37]	**3.22 (2.34 to 4.44)**
Pregnancy related complications	GDM	21	337/3451	8.2 (6.2 to 10.4)	72.6	17/128 [33,47]	60/720 [33,34,40,45]	1.59 (0.96 to 2.64)
	Pre-eclampsia (history or existing)	19	238/3005	7.0 (5.1 to 9.1)	70.9	23/180 [27,33,46]	41/806 [27,33,41,45]	**2.51 (1.55 to 4.08)**
	PROM (including pPROM)	9	128/1184	5.2 (2.4 to 8.7)	71.4	3/64 [27,46,47]	4/40 [31,40,48]	0.47 (0.11 to 1.99)
	IUGR	6	27/903	9.3 (0.0 to 30.7)	97.6	3/23 [26,46]	8/12 [40]	0.20 (0.06 to 0.60)
	Fetal distress	1	4/15	to	-	0	3/12 [31]	Non-estimable
	Placental abruption	2	2/549	0.8 (0.0 to 12.5)	74.0	0	0	Non-estimable

BMI – body mass index, CI – confidence interval, COVID – coronavirus disease, GDM – gestational diabetes mellitus, HTN – hypertension, IUGR – intrauterine growth retardation; MD – mean difference, OR – odds ratio, PROM – premature rupture of membrane, PROM – preterm premature rupture of membrane, SOB – shortness of breath

*Denominator consisted of total number for which that variable was reported.

†Any chronic disease includes all that are listed in the past medical history cell above, except for smoke/tobacco and anemia

‡Cumulative proportion.

§Studies included in severe cases: [24,26-28,31,33-38,43,46,47,49].

‖Studies included in non-severe cases: [24,26-28,30,31,33-38,40-42,44,45,48,50-56].

While almost half (40.6%) had no past medical history, a small proportion of women had anemia (16.1%), hypothyroidism (8.7%), gestational diabetes mellitus (GDM) (8.2%), and pre-eclampsia (7.0%). The risk of having severe COVID-19 was higher among those who had smoked (RR = 3.23; 95% CI = 1.67 to 6.25), chronic cardiac diseases including hypertension (RR = 2.53; 95% CI = 1.68 to 3.81), chronic respiratory diseases including asthma (RR = 2.13; 95% CI = 1.42 to 3.20), and pre-eclampsia (RR = 2.51; 95% CI = 1.55 to 4.08) **Table 2****.**

*Clinical and ICU management:* **Table 3** presents the management of COVID-19 and related complications. The majority of the women were managed with anticoagulants (50.6%) (including heparin, enoxaparin), followed by antibiotics (45.8%) (including azithromycin, vancomycin, ceftriaxone, piperacillin, moxifloxacin, levofloxacin, polymexicin, salbactam, impenam, abipenam, meropenam, sulphanilamide, omidazole, cefazolin, ceftazidime, other quinolone, cephalosporin, and macrolides), hydroxychloroquine (41.3%), antivirals (33.4%) (including oseltamivir, ganciclovir, lopinavir, peramivir, favipiravir, ritonavir, arbidole, ribavirin, interferon), immunotherapy (16.2%), and corticosteroids (14.7%) (including methylprednisolone, dexamethasone, betamethasone). Almost one-quarter were put on oxygen therapy (20.6%).

**Table 3:** Management of COVID-19 and related complications - all cases (n = 31 016)

Characteristics of all pregnant women		Total # of studies	Total events/participants	Pooled proportion (%) (95% CI)	Heterogeneity (I^2%^)
	Antibiotic	14	448/1140	45.8 (27.0 to 65.3)	97.5
	Antiviral	17	514/1742	33.4 (16.3 to 53.1)	98.4
	Hydroxychloroquine	11	305/877	41.3 (21.0 to 63.1)	97.2
	Anticoagulants	5	282/1219	50.6 (12.8 to 88.0)	99.4
	Corticosteroids	8	73/425	14.7 (8.0 to 22.8)	75.5
	Immunotherapy	6	45/827	16.2 (1.7 to 39.7)	96.6
	Plasma therapy	4	183/385	22.5 (0.0 to 79.3)	99.2
	Oxygen therapy	20	321/1812	20.6 (11.0 to 32.2)	96.3
	ICU admission	22	717/27 865	7.2 (4.8 to 9.9)	94.1
ICU management	Mechanical ventilation	20	282/4048	7.7 (3.7 to 12.9)	95.8
	Non-invasive ventilation	5	268/1463	15.1 (3.3 to 32.4)	97.0
	ECMO	6	31/24937	0.3 (0.0 to 1.2)	79.8
	ARDS	4	30/522	10.0 (0.1 to 25.3)	91.6
	Maternal death	14	208/27 680	2.0 (0.8 to 3.8)	94.6

ARDS – acute respiratory distress syndrome, CI – confidence interval, COVID – coronavirus disease, ECMO – extracorporeal membrane oxygenation, ICU – intensive care unit, MODS – multiple organ dysfunction syndrome

About 7.2% of the women were admitted to ICU; of which 7.7% required mechanical ventilation, while 15.1% required non-invasive ventilation (including BiPAP, CPAP) and 0.3% required ECMO. Of all the pregnant women, 30 developed acute respiratory distress syndrome (ARDS) (10%), and 208 (2.0%) died (**Table 3**).

*Pregnancy and perinatal outcomes:* **Table 4** presents pregnancy, birth, and perinatal outcomes. Some of the women experienced spontaneous first-trimester abortion (7.3%), elective termination/induced abortion (2.6%); and fetal loss/miscarriage (2.6%).

**Table 4:** Pregnancy, birth and neonatal outcomes - all cases (n = 31016)

Characteristics of all pregnant women		All cases (n = 31016)				Based on severity (n = 2743)		
**Total # of studies**	**Total events/participants**	**Pooled proportion % (95% CI)**	**Heterogeneity (I^2%^)**	Severe COVID-19* (n = 477)	**Non-severe COVID-19**† **(n = 2449)**	**Risk ratio/Mean difference (95% CI)**
**Pregnancy outcomes**	Spontaneous 1st trimester abortion	5	21/387	7.3 (1.9 to 15.2)	74.1	0	3/61 [36]	Non-estimable
	Elective termination/induced abortion	4	17/540	2.6 (1.4 to 4.3)	65.4	0	4/61 [36]	Non-estimable
	Fetal loss/miscarriage	9	28/874	2.6 (0.5 to 5.6)	66.5	0	14/184 [33,36]	Non-estimable
	Total deliveries	41	2943/4245	80.0 (69.6 to 88.7)	98.2	241/369 [24,26-28,31,33-36,43,46,47,49]	926/1479 [24,26,28,31,33,34,36,40,41,45,48,52,53]	-
	-Vaginal delivery	35	1495/2787	53.8 (46.6 to 60.9)	92.0	53/183 [27,33,35,36,43,46,47]	411/841 [26-28,33-36,40,41,44,45,48,52,53]	**0.59 (0.47 to 0.75)**
	-Caesarean section	35	1256/2740	48.4 (40.6 to 56.3)	93.3	149/207 [27,28,31,33-36,43,46]	439/850 [26,28,31,33,34,36,40,41,45,48,52,53]	**1.39 (1.25 to 1.55)**
**Indication for C-section**	COVID related	10	156/321	49.8 (31.8 to 67.8)	85.9	56/65 [26,27,33,36,47]	66/264 [26,27,33,36]	**3.45 (2.74 to 4.34)**
	Pregnancy related	11	362/520	66.7 (43.3 to 86.8)	95.7	4/33 [33,36,47]	107/273 [26,27,31,33,36]	0.31 (0.12 to 0.78)
**Birth outcomes**	Stillbirths	7	21/1121	1.6 (0.8 to 2.7)	19.8	2/29 [33]	6/126 [26,33]	1.45 (0.31 to 6.81)
	Live births‡							
	-Singleton	14	894/946	96.9 (94.4 to 98.7)	50.6	174/183 [26,27,31,33,38,46,47]	569/602 [26,27,31,33,35,40,41,44,53]	1.01 (0.97 to 1.05)
	-Twins	14	34/1177	1.4 (0.6 to 2.4)	0.2	2/47 [33,46]	15.470 [27,33,35,40,53]	1.33 (0.31 to 5.65)
	Preterm birth <37 weeks	27	539/2393	23.4 (18.9 to 28.1)	83.1	82/182 [27,31,33,35,36,43,46]	78/519 [27,31,33,35,36,41,48]	**4.91 (3.97 to 6.08)**
	Preterm birth <32 weeks	7	45/465	18.2 (0.4 to 38.5)	94.3	34/151 [33,35,46]	8/301 [33,35]	**8.47 (4.02 to 17.85)**
	Low birth weight (<2500 g)	7	107/613	16.6 (11.4 to 22.5)	59.6			-
	Mean birthweight (g)	19		2937.88 ± 87.26	99.6	2274.51 ± 243.62 [27,46]	3077.30 ± 166.93 [27,40,52]	**-802.79 (-826.91 to -778.67)**
**Neonatal outcomes**	Asphyxia	3	74/316	13.2 (1.9 to 31.0)	87.0	1/8 [27]	3/55 [41]	2.29 (0.27-19.44)
	Apgar score at 1 min	9		8.18 ± 0.25	99.4	8.49 ± 0.25 [27,36]	0	Non-estimable
	Apgar score at 5 min	9		9.01 ± 0.15	95.4	8.76 ± 0.19 [27,43,46]	0	Non-estimable
	NICU admission	17	401/1726	23.7 (17.1 to 31.0)	90.2	51/99 [27,33,34,43,46]	57/222 [27,33,34]	**2.01 (1.50 to 2.69)**
	Neonatal deaths	9	24/1161	1.6 (0.4 to 3.3)	58.3	0	0	** to**
**Neonatal COVID-19**	Positive	11	50/1507	3.5 (1.6 to 8.5)	74.6	1/20 [33]	13/205 [26,33,34,45]	**6.69** (**3.46 to 12.94)**

CI – confidence interval, COVID – coronavirus disease, NICU – neonatal intensive care unit

*Studies included in severe cases: [6-28,31,33-38,43,46,47,49].

†Studies included in non-severe cases: [24,26-28,30,31,33-38,40-42,44,45,48,50-56].

Deliveries were reported for 80% of the included women. Of these, 53.8% of the women gave birth vaginally and 48.4% underwent cesarean section. The risk of cesarean birth was 1.39 times higher among severe cases. The majority (66.7%) of cesarean births were performed because of pregnancy-related indications (60.9%), and almost half (49.8%) because of reasons related to COVID-19.

Of all the live births, 96.9% were singleton. Less than a quarter (23.4%) of all births were preterm (<37 weeks) and very preterm (<32 weeks) (18.2%). Almost a quarter (16.6%) of all neonates were low birth weight (LBW) (<2500g), and their mean birth weight was 2937.88g. Almost 13% of the infants experienced asphyxia, and almost a quarter (23.7%) of the newborn was admitted to neonatal ICU (NICU). Their mean Apgar score at 1 minute was 8.18 and at 5 minutes was 9.01. A total of 24 newborns died (1.6%) and 21 were stillborn (1.6%). The risk of preterm births (<37 weeks) (RR = 4.91; 95% CI = 3.97 to 60.8), very preterm birth (<32 weeks) (RR = 8.47; 95% CI = 4.02 to 17.85), and NICU admission (RR = 2.01; 95% CI = 1.50 to 2.69) was higher among mothers with severe COVID-19. Similarly, mean birthweight (MD -802.79; 95% CI = -826.91 to -778.67) was lower in severe COVID-19 cases. Of the total births, 43 newborns were COVID-19 positive (3.5%), and six of these newborn’s SARS-CoV-2 positivity was reported after 12 hours of birth [49].

### Comparative dichotomous studies based on the severity

**Figure 4:**
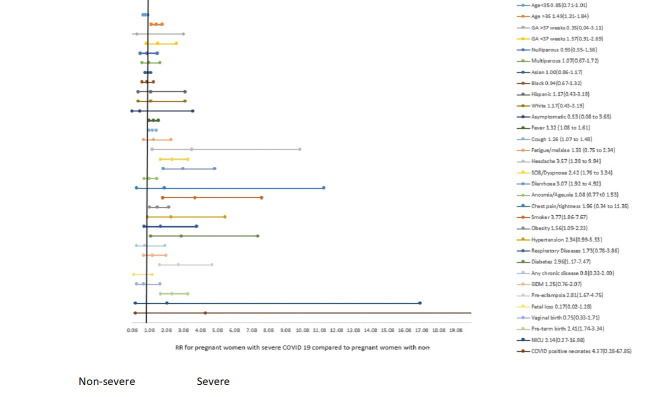
Clinical presentation, risk factors, and pregnancy and perinatal outcomes: pregnant with severe and non-severe COVID-19. The figure presents pooled proportion with 95% confidence intervals using random-effect model.

**Table 5** and **Figure 4** presents the pooled findings from studies that compared non-severe and severe COVID-19 cases from within the same cohort. The comparative pooled analyses showed that women with severe COVID-19 were on average 3.7 years older than women with non-severe COVID-19. Similarly, the risk of having severe COVID-19 among women older than 35 years was 1.49 times higher.

**Table 5:** Studies specifically comparing severe with non-severe COVID-19

Characteristics of all pregnant women		Total # of studies	Severe total events/participants	Non-severe total events/participants	Estimate MD/RR (95% CI)	Heterogeneity (I^2%^)
Demographic	Mean age (years)	6	50	310	3.72 (2.26 to 5.18)	60
	Age <35 years	4	125/208	526/745	0.85 (0.71 to 1.01)	33
	Age >35 years	4	73/208	219/745	1.49 (1.21 to 1.84)	2
	Mean gestational age (weeks)	3	27	60	-2.36 (-6.83 to 2.11)	60
	Asian	3	10/10	135/135	1.00 (0.86 to 1.17)	0
	Black	1	29/75	68/166	0.94 (0.67 to 1.32)	N/A
	White	2	14/83	32/200	1.17 (0.43 to 3.19)	64
	Hispanic	3	34/101	71/214	1.18 (0.58 to 2.39)	77
	Others	2	10/83	26/200	0.93 (0.48 to 1.83)	0
	Third trimester	2	2/2	27/27	1.00 (0.57 to 1.77)	0
	Nulliparous	3	9/21	92/209	0.93 (0.55 to 1.56)	0
	Multiparous	3	9/21	72/209	1.07 (0.67 to 1.72)	0
Presenting symptoms	Asymptomatic	2	0/13	28/187	0.53 (0.08 to 3.65)	0
	Fever	5	31/42	193/325	1.32 (1.08 to 1.61)	0
	Cough	5	37/42	184/328	1.26 (1.07 to 1.48)	23
	Fatigue/malaise	3	18/33	86/205	1.33 (0.75 to 2.34)	38
	Headache	2	9/27	7/123	3.57 (1.28 to 9.94)	0
	SOB/Dyspnoea	5	30/42	55/325	2.42 (1.76 to 3.34)	15
	Diarrhoea	2	7/15	29/191	3.07 (1.92 to 4.92)	0
	Anosmia/Ageusia	2	6/7	77/97	1.08 (0.77 to 1.53)	0
	Chest pain/tightness	2	8/27	18/123	1.96 (0.34 to 11.35)	79
Past Medical history	Mean BMI	2	14	116	4.36 (to 0.67 to 9.38)	92
	Obesity	6	101/237	236/869	1.56 (1.09 to 2.13)	69
	Smoke/tobacco	4	13/146	20/683	3.77 (1.86 to 7.67)	0
	Chronic Resp Disease (incl · asthma)	4	16/156	44/621	1.73 (0.78 to 3.85)	32
	Cardiac Disease (incl · HTN)	3	27/209	36/695	2.34 (0.99 to 5.53)	51
	Diabetes	3	7/142	11/563	2.96 (1.17 to 7.47)	0
Pregnancy related complications	GDM	2	8/27	18/123	1.96 (0.34 to 11.34)	79
	Pre-eclampsia (History or existing)	2	21/136	31/597	2.81 (1.67 to 4.75)	0
Management	Antibiotic	2	26/26	109/122	1.29 (0.63 to 2.67)	89
	Antiviral	2	14/26	57/122	1.87 (1.47 to 2.37)	0
	Steroids	2	7/26	33/122	1.77 (0.86 to 3.65)	0
	Oxygen	4	29/29	12/179	10.20 (1.22 to 85.09)	89
	ICU admission	2	6/22	5/92	11.48 (4.43 to 29.75)	5
Birth and neonatal outcomes	Fetal loss	2	0/137	14/184	0.17 (0.02 to 1.28)	0
	Vaginal births	7	48/159	262/539	0.75 (0.33 to 1.71)	79
	Caesarean births	8	109/155	286/548	1.44 (0.99 to 2.10)	79
	Preterm births (<37 weeks)	5	63/219	69/484	2.41 (1.74 to 3.34)	0
	Mean APGAR score at 1 min	1	7	63	-0.20 (-0.72 to 0.32)	NA
	NICU admission	2	30/67	57/222	2.14 (0.27 to 16.98)	93

BMI – body mass index, CI – confidence interval, COVID – coronavirus disease, GDM – gestational diabetes mellitus, HTN – hypertension, ICU – intensive care unit, IUGR – intrauterine growth retardation; NICU – neonatal intensive care unit, MD: mean difference, RR – risk ratio, SOB – shortness of breath

The risk of severe COVID-19 was not different based on race/ethnicity and parity. However, the risk of severe COVID-19 was higher among those who presented with fever, cough, headache, dyspnoea, and diarrhea. The risk was also higher among those who were obese, had smoked, diabetes, and pre-eclampsia.

Besides the higher proportion of women with severe COVID-19 requiring oxygen and ICU admission, the risk of preterm birth was 2.41 times higher among women with severe COVID-19.

## DISCUSSION

To the best of our knowledge, this is the first systematic evaluation of a large number of pregnant women (n = 31 016) from 62 studies who have been assessed for the clinical presentation, risk factors, and pregnancy and perinatal outcomes based on COVID-19 severity. The review included cases from both referral and community hospitals from 44 countries in Africa, Asia, Australia, Europe, North America, and South America. Of all included pregnant women, 78% presented to hospital during the third trimester; and severity of COVID-19 was presented in only 9% of the studies. Of those, 14% of pregnant women had severe COVID-19.

Results showed that pregnant women with severe COVID-19 were approximately 3.7 years older and the risk of severe COVID-19 was higher among women in a higher age bracket (>35 years). The risk of severe COVID-19 was also higher among women who were obese, had smoked, diabetes, and pre-eclampsia. Chronic hypertension, pre-eclampsia, diabetes, and GDM have been associated with severe COVID-19 in earlier review [9]; while co-morbidities have been identified as an explanatory variable for maternal admission to ICU [14]. While almost half of all women were asymptomatic, fever, cough, fatigue, and anosmia/ageusia were the most commonly reported symptoms. Similar findings have been reported in other reviews [9-11,14]. Around half of all women were given antibiotics, anticoagulants, and hydroxychloroquine, one in three were given antivirals, and nearly one in five were managed with corticosteroids. Of all included pregnancies complicated with COVID-19 infection, 7% were admitted to ICU, 8% required mechanical ventilation, 15% non-invasive ventilation, and 0.3% ECMO. Almost half of all births occurred via cesarean section; one-quarter all births were preterm, less than one-fifth had LBW, and a quarter was admitted to NICU. However, it was not always reported in the studies if babies in need of intensive care were admitted to NICU or for isolation purposes and therefore the results could be misleading. Earlier systematic compilation of published evidence also reported similar findings [14,57,58]. Our review found lower rates of maternal (2.0%) and neonatal (1.6%) mortality and the findings are consistent with earlier reviews in the domain [9,14,57,58]. Neonatal SARS-CoV-2 positivity was small (3.5%) and consistent across other evidence synthesis [57,58].

Although it is a comprehensive assessment of all COVID-19 infected pregnant women, the review itself and the studies included in the review have several limitations. The findings of this review should be cautiously interpreted mainly because: first, the review included case series that brings an inherent bias due to study design, however, on quality assessment majority of these studies were rated high. Second, the majority of women (87.1%) included in the review presented to hospitals during the third trimester, therefore we could not compare the clinical presentation in early vs late pregnancy. Third, approximately one-third of all women were asymptomatic which may be because of studies on reported universal screening for SARS-CoV-2 in women admitted for delivery. The inclusion of asymptomatic women from the universal screening may have also influenced better maternal and perinatal outcomes. Fourth, the majority of the socio-demographic and baseline health measures were not uniformly and consistently reported in all included studies; and therefore, some of the risks are very high with wider CIs because of the smaller sample size for some of the outcomes. Similarly, not all studies categorized and reported separate outcome for women infected with severe and non-severe COVID-19; therefore, results based on severity was generated from a sub-group of women included. Fifth, the indication for cesarean section was not clearly stated and it is quite possible that the decision was influenced by the anxieties and perplexities around the potential consequences of the novel coronavirus. In near future, studies around the implications of COVID-19 in pregnancy may contain thorough and complete information about both maternal and fetal conditions at the time of delivery and the rationale behind obstetrical interventions. Sixth, the review identified fewer cases from the African regions. Seventh, most of the included studies reported on advance maternal age and obesity, therefore it was easy to look at their association with COVID-19 severity. However, as more studies from low-income settings are published, it would be interesting to learn the association of underage (<25 years) and underweight (BMI<18.5) pregnant women and the presentation of COVID-19 and its severity. Eighth, although we tried our best not to include the same case twice, there is a possibility that the case is reported more than once. Ninth, we couldn’t carry out multivariable analysis to identify factors associated with severe COVID-19 among pregnant women after adjusting for socio-demographic and health profile of the women. However, in the future, if data are presented at the patient level then individual patient data meta-analysis (IPD-MA) would be an ideal approach to providing insights into recognizing and managing high-risk pregnant women. Lastly, the overall findings from this review should be interpreted with caution and the data should be constantly updated as the global COVID-19 scenario unfolds.

The results of the review indicate that the risk of severe COVID-19 is higher among pregnant women with risk factors and pre-existing co-morbidities, and the risk of poor pregnancy and perinatal outcomes of those women with severe COVID-19 are also higher. This is an area of concern as the COVID-19 pandemic is spreading around the world and most likely many pregnant women will be affected [59]. It is, therefore, important to identify high-risk women and routinely check all women coming for prenatal visits for COVID-19. In this pandemic, it is also very vital to systematize screening for all pregnant women visiting inpatient or outpatient facilities; and manage all suspected/confirmed COVID-19 cases and prepare maternity wards in the best possible way. Although our results indicate that the risk of vertical transmission is very low, it is important to institute appropriate protective measures and hygiene procedures during delivery. Mothers should be advised to wear a medical mask during any contact with the baby such as during breastfeeding, skin-to-skin care, and should be supported to share a room with the baby. Management of pregnant women and babies infected with COVID-19 should be performed following national or international guidelines.

## CONCLUSIONS

This review provides information required to guide current care during this pandemic and health care providers may consider these findings for effective management of pregnant women infected with COVID-19 that would reduce pregnancy-related adverse consequences including maternal and newborn morbidity and mortality. At the same time, it is important to interpret the findings cautiously and update them constantly as the pandemic unfolds. Studies from Denmark and Ireland have reported falling numbers in premature and very and extremely LBW babies particularly those born before 28 weeks in many parts of the world by startling 70 to 90 percent [60,61], our review only depended upon reported case-series and smaller population data that did not report on falling numbers of preterm births in the population. While, these findings are counterintuitive and researchers have speculated an association with strict lockdown and isolation; whereby women may have experienced less stress from work and commute, more support from families at home, and less exposure to air pollution and infections in general (such as influenza). Although the finding pertains to a general reduction in the overall rates of premature births and not necessarily implies to women infected with COVID-19, but such elusive observations are providing directions for future research. More coalitions are built for better prospective data collection and international surveillance and a few of these efforts include the WHO Collaborative and International Perinatal Outcomes in the Pandemic (iPOP).

## Additional material

Online Supplementary Document
