# Supplementary material for: A systematic review and meta-analysis of data on pregnant women with confirmed COVID-19: Clinical presentation, and pregnancy and perinatal outcomes based on COVID-19 severity
Source: J Glob Health. 2021 Jun 30;11:05018. doi: 10.7189/jogh.11.05018 (PMC8248750; doi:10.7189/jogh.11.05018)
Supplement: Online Supplementary Document [file jogh-11-05018-s001.pdf]

Supplementary table 1: Logic Grid: COVID-19 and Pregnancy outcomes

Covid	Pregnancy
Coronavirus[MeSH] OR COVID-19[MeSH] OR “severe acute respiratory syndrome coronavirus 2”[MeSH] OR<br><br>Covid[All Fields] OR Covid19[All Fields] OR Covid-19[All Fields] OR “covid 19”[All Fields] OR “corona”[All Fields] OR "2019 nCoV”[All Fields] OR 2019nCoV[All Fields] OR "2019 novel coronavirus"[All Fields] OR "COVID 19"[All Fields] OR "SARS CoV-2"[All Fields] OR "new coronavirus"[All Fields] OR "novel coronavirus"[All Fields] OR (Wuhan AND coronavirus)[All Fields] OR "SARS-CoV"[All Fields]	Pregnancy[MeSH] OR “pregnant women”[MeSH] OR Pregnancy OR pregnant OR Pregnan*

Supplementary table 2: Case reports

Study Name	Country	Exact data of case reported	Reason for exclusion
Zuzana Kolkova 2020 [1]	Malmo, Sweden	Not reported	Case-report
Yassmine Abourda 2020 [2]	Morocco	Not reported	Case-report
Suzanne Slayton-Milam 2020 [3]	Washington, USA	Not reported	Case-report
S. O. Cheng 2020 [4]	London, UK	April 5, 2020	Case-report
David A. Forero-Pena 2020 [5]	Venezuela	March 28, 2020	Case-report
R Polónia-Valente 2020 [6]	Porto, Portugal	March 17, 2020	Case-report
Bianca Pulinx 2020 [7]	Belgium	Not reported	Case-report
Molly C Easterlin 2020 [8]	Los Angeles, USA	Not reported	Case-report
Antonella Ferrariolo 2020 [9]	Genoa, Italy	Not reported	Case-report
Ying Yu 2020 [10]	Wuhan, China	Jan 28, 2020	Case-report
Harsh Mehta 2020 [11]	New Jersey, USA	Not reported	Case-report
Irshad Ahmed 2020 [12]	Birmingham, UK	April 8, 2020	Case-report
Katy Kuhurt 2020 [13]	London, UK	Not reported	Case-report
De Socio GV 2020 [14]	Perugia, Italy	March 30, 2020	Case-report
Ida Martinelli 2020 [15]	Milan, Italy	Mar 29, 2020	Case-report
Victoria Vallejo 2020 [16]	New York, USA	Not reported	Case-report
Christine Blauvelt 2020 [17]	San Francisco, USA	Not reported	Case-report
Leah Hong 2020 [18]	Detroit, USA	Not reported	Case-report
Dawei Lu 2020 [19]	Wuhan, China	Feb 11, 2020	Case-report
Joana Lyra 2020 [20]	Porto, Portugal	Nor reported	Case-report
David Baud 2020 [21]	Lausanne, Switzerland	March 20, 2020	Case-report
Jianwei Li 2020 [22]	Zhongshan, China	Jan 28, 2020	Case-report
D Gonzalez Romero 2020 [23]	Spain	Not reported	Case-report
Haifa Xia 2020 [24]	Wuhan, China	Jan 20, 2020	Case-report
Paul C Browne 2020 [25]	South Carolina, USA	July 7, 2020	Case-report
Shuming He 2020 [26]	Zhongshan, China	Feb 2020	Case-report
E Kalafat 2020 [27]	Ankara, Turkey	March 20, 2020	Case-report
Parisa Karami 2020 [28]	Iran	Not reported	Case-report
Zinggui Liao 2020 [29]	China	Feb 9, 2020	Case-report
Mengdie Li 2020 [30]	Xinyang, China	Not reported	Case-report
Li Yao 2020 [31]	Hefei, China	Feb 11, 2020	Case-report
Siying Zhuang 2020 [32]	Wuhan, China	Jan 20, 2020	Case-report
Rong Wen 2020[33]	China	Feb 4, 2020	Case-report
Lysien Zambrano 2020[34]	Honduras	Mar 9, 2020	Case-report
Xiali Xiong 2020[35]	Beijing China	Jan 29, 2020	Case-report
Maria Claudia Alzamora 2020 [36]	Lima, Peru	March 29, 2020	Case-report
Zhoujie Peng 2020 [37]	Chongqing, China	Not reported	Case-report
Dong Hwan Lee 2020 [38]	Republic of Korea	Jan 19, 2020	Case-report
Yang Li 2020 [39]	Zhejiang, China	Feb 6, 2020	Case-report
Shaoshuai Wang 2020 [40]	Wuhan, China	Feb 1, 2020	Case-report
Xiaotong Wang 2020 [41]	China	Feb 2, 2020	Case-report
Sebastian Gidlof 2020 [42]	Stockholm, Sweden	Not reported	Case-report
Sara Iqbal 2020 [43]	USA	Not reported	Case-report
Ane Cecilie Munk 2020 [44]	Norway	Early April 2020	Case-report
Christian Chigozie Makwe 2020 [45]	Lagos, Nigeria	Not reported	Case-report
Kelly Elizabeth Kinsey 2020 [46]	New York, USA	Not reported	Case-report
Rita Figueiredo 2020 [47]	Porto, Portugal	Not reported	Case-report
Mariam Naqvi 2020 [48]	Los Angeles, USA	Not reported	Case-report
Jing Peng 2020 [49]	Wuhan, China	Jan 23, 2020	Case-report
Yantian Lv 2020 [50]	Wuhan, China	Feb 6, 2020	Case-report
Rong Wen 2020 [51]	Qingdao, China	Jan 22, 2020	Case-report
Jin-Gon Bae 2020 [52]	Korea	Feb 12, 2020	Case-report
Alejandro Garcia Rodriguez 2020 [53]	Leon, Spain	Not reported	Case-report
Tayebeh Rashidian 2020 [54]	Ilam, Iran	Not reported	Case-report
Moti Gulersen [55]	New York, USA	Not reported	Case-report
Priya Patel [56]	USA	Not reported	Case-report
Alex Mark [57]	USA	Not reported	Case-report
Yekta Parsa [58]	Tehran, Iran	Between March to April 2020	Case-report
Sofiane Kouas [59]	Mahdia, Tunisia	Early March 2020	Case-report
M.L. Rodrigues [60]	Amadora, Portugal	Not reported	Case-report
Anne-Sophie Michel [61]	Paris, France	Not reported	Case-report
Claire M. McCarthy [62]	Dublin, Ireland	Not reported	Case-report
Muhammad Suleman Rana [63]	Islamabad, Pakistan	June 4, 2020	Case-report
Talal Hamood Alwardi [64]	Muscat, Oman	Not reported	Case-report

Supplementary table 3: Reasons for excluding studies

Study Name	Country	Exact data of case reported	Reason for exclusion
Ardi Hirshberg 2020 [65]	Philadelphia, USA	Not reported	Overlap with other study
Patricia Barbero 2020 [66]	Madrid, Spain	March 3 to May 31	Overlap with other study
Desmond Sutton 2020 [67, 68]	New York, USA	22 March to April 4, 2020	Overlap with other study
Maria Andrikopoulou 2020[69]	New York, USA	Mar 13 to Apr 18, 2020	Overlap with other study
Zhiqiang Wang 2020[70]	Wuhan, China	Dec 8 to April 1 2020	Overlap with other study
Na Li 2020 [71]	Wuhan China	Jan 24 to Feb 29, 2020	Overlap with other study
Rong Chen 2020 [72-74]	Wuhan, China	30 Jan to 24 Feb 2020	Overlap with other study
Dehan Liu 2020 [73-75]	Wuhan China	Jan 20 to Feb 10, 2020	Overlap with other study
Huanhuan Liu 2020 [76]	Hubei, China	Jan 27 to Feb 14, 2020	Overlap with other study
Pu Yang 2020 [77]	Wuhan, China	Jan 20 to Jan 29, 2020	Overlap with other study
Noelle Breslin 2020 [78, 79]	USA	March 13 to 27, 2020	Overlap with other study
Huijun Chen 2020[80]	Wuhan China	Jan 20 to Jan 31, 2020	Overlap with other study
Di Lei 2020 [81]	Wuhan, China	Jan 22 to Feb 1, 2020	Overlap with other study
Siyu Chen 2020[82]	Hubei, China	Jan 20 to Feb 10, 2020	Overlap with other study
Yan Chen 2020[83]	Wuhan, China	Not reported	Overlap with other study
Cuifang Fan 2020[84]	Wuhan, China	Jan 17 to Jan 23, 2020	Overlap with other study
Chen Shou 2020[85]	China	Till feb 25	Overlap with other study
Nan Yu 2020[86]	Wuhan, China	Jan 1 to Feb 8, 2020	Overlap with other study
Huaping Zhu 2020[87]	Hubei, China	Jan 20 to Feb 5, 2020	Overlap with other study
Hui Yang 2020[88]	Wuhan, China	20 Jan to Mar 5, 2020	Overlap with other study
Chunchen Wu 2020[89]	Wuhan, China	Jan 23, Feb 10	Overlap with other study
Dongmei Cao 2020[90]	Wuhan, China	Jan 23 to Feb 23, 2020	Overlap with other study
Enrico Ferrazi 2020[91]	Northern Italy	Mar 1-20, 2020	Overlap with other study
Y Wu 2020[92]	Wuhan, China	31 Jan to 9 Mar, 2020	Overlap with other study
A Govind 2020[93]	London, UK	Mar 7 to Apr 22, 2020	Overlap with other study
Wei Liu 2020[94]	Wuhan, China	Jan 31 to Feb 29, 2020	Overlap with other study
Hui Yang 2020 [95]	Wuhan, China	Jan 20 to Mar 19, 2020	Overlap with other study
William R Cooke 2020 [96]	Reading, UK	Not reported	Overlap with other study
YingChun Zeng 2020[97]	Hubei, China	Until Feb 16, 2020	Overlap with other study
Nathan S Fox 2020[98]	NY, USA	Mar 20 to Apr 30, 2020	Overlap with other study
S Chen 2020[99]	Wuhan, China	Feb 4, 2020	Overlap with other study
Alexandre J Vivanti 2020 [100]	Paris, France	March 12 to April 13, 2020	Overlap with other study
LinLi Yue 2020 [101]	Hubei, China,	Jan 24 to Feb 29	Overlap with other study
Xiaolin Hu 2020 [102]	Wuhan, China	Jan 20 Feb 20, 2020	Overlap with other study
Matthew J. Blitz [103, 104]	New York USA	March 1, May 6, 2020	Overlap with other study
Lu Zhang 2020 [105]	Wuhan China	Jan 30 o Mar 1 2020	Overlap with other study
Shuang Xu 2020 [106]	Wuhan, China	Jan 15 to March 15	Overlap with other study
Shaoshauai Wang 2020 [107]	Wuhan, China	Jan 19, March 2, 2020	Overlap with other study
Yanfen Chen 2020 [108]	Wuhan China	Jan 20 to Feb 29, 2020	Overlap with other study
Yan-Ting Wu 2020 [109]	Wuhan China	Jan 30 to Mar 10, 2020	Overlap with other study
Biheng Cheng 2020 [110]	Wuhan, China	Jan 15 to Feb 23	Overlap with other study
Miguel A. Marín Gabriel 2020 [111]	Madrid, Spain	March 13, March 29, 2020	Overlap with other study
Lina Antoun 2020 [112]	Birmingham, UK	Feb 2020 to April 2020	Overlap with other study
Jeffery Perlman 2020 [113]	New York, USA	March 22 to April 15, 2020	Overlap with other study
Lingkong Zeng 2020 [114]	Wuhan, China	Jan to Feb 2020	Overlap with other study
Min-Zhu Yin 2020 [115]	Wuhan, China	Jan 28 to Feb 28, 2020	Overlap with other study
Pin Liu 2020 [116]	Wuhan, China	Jan 20 to March 3, 2020	Overlap with other study
Renbin Zhou 2020 [117]	Wuhan, China	Not reported	Overlap with other study
Wei Liu 2020 [118]	Wuhan, China	Jan 7 to March 4, 2020	Overlap with other study
Dilek Sahin [119]	Ankara, Turkey	March 11 to June 11, 2020	Overlap with other study
Kate R. Woodworth 2020 [120]	USA (16 jurisdictions)	March 29 to Oct 14, 2020	Overlap with other study
Chelsea A DeBolt 2020 [121]	NY & Philadelphia, USA	March 12 to May 5, 2020	Overlap with other study
Rong Yang 2020 [122]	Wuhan, China	Jan 13 to March 18	Overlap with other study
Miranda J Delahoy 2020 [123]	USA (13 jurisdictions)	March 1 to Aug 22, 2020	Overlap with other study
Lakshmi Panagiotakopoulos 2020 [124]	USA (8 jurisdiction)	March 1 to May 30, 2020	Overlap with other study
Mario Isaac Lumbreras-Marquez 2020 [125]	Mexico	As of May 17, 2020	Overlap with other study
Ellington 2020 [126]	United states	Jan 22 to June 17	Overlap with other study
Ozsurmeli M 2021 [127]	Istanbul, Tukey	Mar 11 to July 01	Overlap with other study
RJ Martinezz-Portilla 2020 [128]	Mexico	Feb 1 to 28 Oct 2020	Overlap with other study
Sheela Maru 2020 [129]	New York, USA	March 29, April 22, 2020	Overlap with other study
Miguel A Marin Gabriel 2020 [130]	Spain	March 13 to May 31, 2020	Overlap with other study
Monica Cruz-Lemini 2021 [131]	Spain	March to May 2020	Overlap with other study
Yuping Guo 2020 [132]			
Daigo Ochiai 2020 [133]	Tokyo, Japan	Apr 6 to Apr 27, 2020	Case series of less than 10 cases (n=2)
Wen-Han Chang [134]	Taiperi, Taiwan	Not reported	Case series of less than 10 cases (n=2)
Jing-yi Tang 2020 [135]	Wuhan, China	23 Jan and 28 Jan 2020	Case series of less than 10 cases (n=2)
Dongmeri Cao 2020 [136]	Wuhan China	Feb 8 and Feb 26, 2020	Case series of less than 10 cases (n=2)
A.Gheysarzadeh 2020 [137]	Ilam, Iran	Not reported	Case series of less than 10 cases (n=4)

Helena Lucia Barroso dos Reis 2020 [138]	Espírito Santo, Brazil	Not reported	Case series of less than 10 cases (n=3)
Danilo Buonsenso 2020[139]	Rome, Italy	Not reported	Case series of less than 10 cases (n=7)
Serafina Perrone 2020 [140]	Parma, Italy	March to April 2020	Case series of less than 10 cases (n=4)
Luigi Gagliardi 2020 [141]	Pisa, Italy	Upto April 19, 2020	Case series of less than 10 cases (n=3)
Matthew P. Romagano 2020 [142]	New Jersey, USA	March to Apr 2020	Case series of less than 10 cases (n=8)
Alexandra Jusssela 2020 [143]	New Jersey, USA	March 2020	Case series of less than 10 cases (n=2)
Elizabeth Lucarelli 2020 [144]	New Jersey, USA	March 31 to May 20, 2020	Case series of less than 10 cases (n=3)
Mingyang Sun 2020 [145]	China	Not reported	Case series of less than 10 cases (n=3)
Suliman Khan 2020 [146]	Wuhan, China	Jan 15 to Mar 15, 2020	Case series of less than 10 cases (n=3)
Xu Chen 2020 [147] Abstract only	China	Jan 19 to Feb 10, 2020	Case series of less than 10 cases (n=3)
Wenhui Huang 2020 [148]	Wuhan, China	Jan 24 to Feb 19, 2020	Case series of less than 10 cases (n=8)
Tingting Zheng 2020 [149]	Yichang city China	Jan 20 to April 9, 2020	Case series of less than 10 cases (n=2)
Sedigheh Hantoushzadeh 2020 [150]	Tehran, Iran	Mid Feb to Mid-March 2020	Case series of less than 10 cases (n=9)
Nicole Olivini [151]	Rome, Italy	Early March	Case series of less than 10 cases (n=5)
Julia Fashner [152]	USA	As of April 29, 2020	Case series of less than 10 cases (n=9)
Yu Chen 2020 [153]	Shenzen, China	Not reported	Women were suspected and were negative on test
Beatrice Tasssis 2020 [154]	Milan, Italy	April 1 to April 9, 2020	The study only provided the number of women came positive on COVID on universal screening
Asma Khalil 2020 [155]	London, UK	As of April 20, 2020	Only reported on universal screening
Erika Molteni 2020 [156]	UK, Sweden, and USA	Not reported	The study included web-based population surveys results on information on participants recall
Nina le Cour Freiesleben 2020 [157]	Copenhagen, Denmark	Feb 17 to April 23, 2020	Study reported suspected and confirmed COVID-19
Stefano Cosma 2020 [158]	Turin, Italy	Feb 22 to May 21, 2020	Study compared first trimester abortion with ongoing pregnancy and reported IgG and IgM of the participants.
Weiyong Liu 2020 [159]	Wuhan, China	Up until Feb 13, 2020	The study discussed the diagnostic procedures using placental, vaginal, breast and oropharyngeal swabs
Yuanyuan Dong 2020 [160]	Shanghai, China	Up until Feb 8, 2020	The participants were pregnant but their age ranged between 14 and 79 years. Study also reported 79 years old pregnant women
Claudia Massoratti 2020 [161]	Genoa, Italy	April 1 to April 30, 2020	The study only provided the number of women came positive on COVID on universal screening
Rakesh Waghmare 2020 [162]	Maharashtra, India	25 April to May 20, 2020	The study only provided the number of women came positive on COVID on universal screening and the number who were asymptomatic and symptomatic

Supplementary table 4: Characteristics of included studies

Study and year	Study design	Country and time period	Setting	Total number	Baseline characteristics of women			Presenting signs and symptoms of all pregnant women	Maternal and neonatal COVID-19 status	Delivery characteristics in COVID-19 positive women	Management of pregnant women with COVID-19	Maternal pregnancy /perinatal outcomes of COVID-19 positive women
					Mean age (yrs)	Mean gestational age (wks)	Past medical history					
Gabriele Saccone 2020<br><br>WAMP study [163, 164]	Prospective	**22 countries**	Multicounty and multicentre	N=388	32.2±6.1	30.6 ± 9.5	Obesity = 28/388	Asymptomatic = 94/388<br>Fever =171/388<br>Cough = 202/388<br>Rhinorrhoea = 29/388<br>Mylagia = 56/388<br>Anosmia = 21/388<br>SOB = 60/388<br>Diarrhoea = 16/388<br>Conjunctivitis = 9/388	History travel or meeting infected case = NR<br>**COVID confirmed women**<br>Maternal death = 3/388<br>Maternal COVID +ve 388<br>Neonates COVID –ve 1/250	Discharged with ongoing pregnancies = 123/388<br>Total deliveries = 265/388<br>Abortion =6/31<br>Termination of pregnancy = 3/265<br><br>Caesarean deliveries = 136/250	Hydroxychloroquine = 90/388<br>Antibiotics (Azithromycin)= 79/388<br>Antiviral (oseltamivir, darunavir, cobicistat, lopinavir, ritonavir, remdesivir) = 72/388<br>ICU = 43/388<br>Intubation =25/388<br>ARDS = 7/388<br>ECMO = 2/388	Stillbirth = 6/265<br>Neonatal death = 5/250<br>Perinatal death = 11/265<br>NICU admission = 69/250<br>Birthweight = 2921±772<br>LBW 52/250<br>IGR = 10/265<br>Preterm birth = 70/265
InterCOVID 2020 [165]	Prospective	**18 countries**<br><br>March to April 16	Multicountry and multicentre	N= 672	30.2 + 6.1	NR	Smoker = 18<br>Diabetes = 32<br>Overweight = 317<br>Thyroid = 71<br>Cardiac disease = 12<br>Hypertension = 27<br>Respiratory disease = 26<br>Kidney disease = 5<br>Malaria = 1.7<br>Tuberculosis = 0.5%<br>Mean BMI = 25.7 + 5.8<br>GDM = 76<br>PIH = 57<br>Anaemia = 131<br>PROM 108<br>Fetal distress = 80	Chest pain = 18<br>Diarrhea/vomiting = 45<br>Limk or joint pain = 49<br>Sore throat = 68<br>Flu like symptoms = 75<br>Runny nose = 78<br>Headache = 85<br>Breathlessness = 85<br>Tiredness/lethargy = 107<br>Loss of smell = 113<br>Fever = 193<br>Cough = 236<br>Asymptomatic = 269	**Laboratory COVID confirmed women**<br>Maternal death = 10<br>Neonates positive for COVID-19 = 45	Caesarean section = 319	Anticoagulant = 73<br>ICU admission = 53	Preterm birth = 25<br>Birthweight = 2960 + 690<br>LBW = 142<br>SGA = 13.5%<br>Stillbirth = 6<br>Neonatal mortality = 12
Mariam Knight 2020 [166, 167]<br><br>UKOSS	Cohort	UK<br><br>Mar 01 to Apr 29, 2020	UK Obstetric Surveillance System from 194 hospitals	N=427	<20 = 4<br>20-34 = 248<br>>35 = 175<br><br>Asian =103/427<br>Black = 90/427<br>Chinese = 30/427<br>Mixed = 10/427	Median 34 weeks (29 to 38 weeks)	Smoker = 20/427<br>Obese = 140/427<br>Overweight = 141/427<br>Asthma = 31/427<br>Hypertension = 12/427<br>Cardiac disease = 6/427<br>Diabetes = 3/427<br>GDM = 50/427	Fever = ~285<br>Cough = ~240<br>Breathlessness =~155<br>Fatigue or lethargy = ~60<br>Headache = ~55<br>Joint pain = ~50<br>Sore throat = ~45<br>Vomiting = ~45<br>Diarrhoea = ~20<br>Rhinorrhoea = ~20	History travel or meeting infected case = NR<br>**Non- Severe COVID-19 in mother = 386/427**<br>**Severe COVID-19 in mother = 41/427**<br>Maternal COVID +ve 427/427nasopharyngeal swab for PCR<br>Neonates COVID +ve 12/265 on nasopharyngeal swab RNA<br>**Maternal death = 5/427 (severe =3)**<br>Discharged = 397/427	Discharged with ongoing pregnancies = 161/427 (severe =8)<br>Miscarriage = 4/427<br>Total deliveries = 266/427 (Severe = 33)<br>Caesarean section = 159/262<br>Vaginal = 106/262<br>Indication for C-section = COVID = 42/262<br>Pregnancy complication = 114/262	Antiviral (oseltamivir, lopinavir/ritonavir) = 9/427<br>Corticosteroid (FLM) = 64/427<br>Intubated = 18/427<br>ICU = 41/427	Livebirths = 259 (Singleton 247 + 6 twins)<br>Preterm births = 66/262<br>Stillbirths = 3/262<br>Neonatal death = 2/259<br>NICU admission = 67/265
Mariano Doria 2020 [168]<br><br>12 cases separately reported<br><br>**Universal screening**	Cohort	Portugal<br><br>Mar 25, Apr 15, 2020	Unidade Local de Matosinhos/Pedro Hispano Hospita	N=103<br><br>Positive = 12	31.91±535<br><br>*Age separately reported	37.5 ± 2.9<br><br>* separately reported	Ulcerative colitis = 1/12<br>Psoriasis =1/12<br>Sever scoliasis =1/12<br>Behcet Syndrome =1/12<br>Myopia=1/12<br>Chronic hypertension =1/12<br>Asthma=1/12<br>Raynaud syndrome =1/12<br>GDM=1/12<br>PROM =1/12<br>None= 6/12	Asymptomatic =11/12<br>Headache = 1/12	History travel or meeting infected case = NR<br>**Non- Severe COVID-19 in mother = 12/103**<br>Maternal COVID +ve 11/103 nasopharyngeal swab for PCR<br>Neonates COVID –ve 11/11 on nasopharyngeal swab for PCR	Discharge with ongoing pregnancy =2/12<br>Total deliveries =10<br>Caesarean section = 6/10<br>Vaginal deliveries =4/10	NR	Births = 11 (9 singleton, 1 twin)<br>Birthweight = 2690.90 ± 336.47 * separately reported<br>Apgar score at 1 min = 8.90 ± 0.30 * separately reported<br>Apgar score at 5 min = 9.90 ± 0.30 * separately reported<br>IUGR=8/11
Maira LS Takemoto [169]	Prospective	All over Brazil	All over Brazil	N=978	32+5<br>30+5	NR	Asthma = 22<br>Cardiovascular = 117<br>Diabetes = 86<br>Obesity = 43	NR	Lab confirmed = 950<br><br>Maternal death = 124	NR	ICU admission = 207<br>Invasive ventilation = 98<br>Non-invasive = 219	NR

Brazillian ARDS-SS		Until June 18, 2020			1st trim = 7<br>2nd tri = 157<br>3 tri = 495<br><br>White = 235<br>Black = 51<br>Yellow = 3<br>Brown = 451<br>Indigenous = 6		Any comorbidity = 273					
NethOSS 2020 [170]	Registry	Netherland<br><br>March 1 to July 31	Netherland Registry	N=159	NR	NR	Fetal distress = 1/90	Cough = 72<br>Fever = 60<br>SOB = 42	Neonates COVID +-ve = 93<br><br>**Maternal death = 1**	Caesarean section = 29/90	Antibiotics = 36<br>Antiviral (Remdesevir) = 3<br>Hospitalization = 106<br>Oxygen = 28<br>Intubation = 4	Livebirths = 93 (87 singleton, 3 twin)<br>Preterm birth = 90<br>Miscarriage = 2/94<br>NICU admission = 21<br>Neonatal death = 0
Francesca Crovetto 2020 [171]	Cohort	Barcelona, Spain<br>April 14 to May 5	Hospital Sant Joan de Déu and Hospital Clínic	N= 125	33.7 + 5.1 /54<br>31.8 + 6.1 / 71<br><br>White = 74	First trimester = 54<br>Third trimester = 71<br>Nulliparous = 70<br>Twin pregn = 3	Chronic hypertension = 3<br>Diabetes= 1<br>Authoimmune disease = 2<br>Respiratory disease = 5<br>Smoking = 10<br>Mean BMI = 24.1 + 6.2/54; 23.9 + 4.3/71	Asymptomatic = 75<br>Fever = 23<br>Cough = 22<br>Dyspnoea = 8<br>Tiredness/fatigue = 9<br>Dore throat = 9<br>Rash = 0<br>Diarrhoea = 9<br>Anoemia/agusua = 23	**Non- Severe COVID-19 in mother = 122**<br>**Severe = 3 oxygen**	NR	Oxygen therapy = 3	NR
Manel Mendoza 2020 [172]<br><br>Separate results for severe and non-severe cases	Perspective	Barcelon, Spain<br><br>Mar 13 tp Apr 10, 2020	Hospital Universitari Vall d'Hebron, Universitat Autònoma de Barcelona	N=42	32<br><br>White 52%<br>Latin American 40%<br>Others 7%	32 (IQR 26 to 37.5)<br><br>Severe = 30+0, 22+6, 37+5, 36+4, 32, 20+3, 27+4, 20+1	Smoking = 2 (1 severe, 1 non-severe)<br>Preeclampsia history =0<br>Hypertension = 0<br>Chronic kidney disease = 0	NR	No History travel or meeting infected case<br>**Non- Severe COVID-19 in mother=78**<br>**Severe COVID-19 in mothers = 8**<br>Maternal COVID +ve on oro-pharyngeal swab for PCR	NR	ICU admission = 8/42	NR
Rafael San-Juan 2020 [173]<br><br>Based on severity	Cohort	Madrid, Spain<br><br>March 5, Apr 5, 2020	Department of Obstetrics of the University Hospital "12 de Octubre"	N=32	32 ±7<br><br>Hispanic = 21<br><br>Severe = 39 + 28 + 33 + 38 + 40 + 37	29.2± 2.17<br><br>First trimester = 1<br>Second trimester = 9<br>Third trimester = 22	No comorbidity = 26/ 32<br>Asthma = 4/32<br>Obesity = 1/32<br>Multiple sclerosis = 1/32<br>GDM = 2/32	Cough = 31/32<br>Dyspnoea = 25/32<br>Fever = 20/32<br>Fatigue = 15/32<br>Myalgia = 13/32<br>Headache = 9/32<br>Coryza = 5/32<br>Chest pain = 4/32<br>Diarrhoea = 1/32<br>Anosmia/dysgueia = 1 /32	**Non-severe COVID-19 in mothers = 14**<br>**Severe COVID-19 in mother = 18**<br><br>Maternal COVID +ve = 32	NR	Hospital admission = 29<br>Antiviral = 8<br>Hydroxychloroquine = 22<br>Antibiotic = 23<br>Immunomodulatory = 5<br>Corticosteroid = 3<br>Plasma therapy = 2<br>Oxygenation = 18<br>ICU admission = 2<br>ARDS = 8<br>Mortality = 0	NR
Augusto Pereira 2020[174]	Restrospective	Madrid, Spain<br><br>March 14 to April 14	Puerta de Hierro University Hospital Madrid	N=60	<30 years = 11<br>30-34 = 24<br>35-40 = 22<br>>40 = 3	First trimester = 10 (1-12)<br>2 trimesters = 13-26 = 16<br>3 trimesters = 27-41 = 34	Preeclampsia = 3<br>DVT = 2	Asymptomatic = 15/60<br>Fever = 34/60<br>Cough = 34/60<br>Dyspnoea = 17/60	History travel or meeting infected case = 28/60<br>Maternal COVID +ve = 60 on oro-pharyngeal swab for PCR<br>**Non-Severe COVID-19 in mothers = mild = 9/60**<br>**Moderate = 7/60**<br>**Severe = 7/60**<br>Neonates COVID –ve = all on PCR	Discharged with ongoing pregnancy = 37<br>Total deliveries = 23<br>Vaginal delivery = 14/23<br>Instrumental delivery = 4/23<br>Caesarean section = 5/23	Hydroxychloroquine = 39/60<br>Antiviral = 11/60	Preterm birth = 2/23
Oscar Martinez-Perez 2020 [175]	Prospective	Spain	Multicentre 3 maternity hospitals in Spain	N=82	Median 35, 33 and 36	Median 39, 38, 29	GDM = 1/78 severe 0<br>Pre-eclampsia = 3 / 78 severe ¼	Asymptomatic = 22/78	No History travel or meeting infected case<br>**Non- Severe COVID-19 in mother=78/82**	Total deliveries=82<br>Caesarean births = 37/78, severe 4/4<br>Vaginal deliveries = 41/78	Oxygen = 11/78, severe 4/4	Preterm birth = 1414/78 and 1/4 severe<br>Very preterm = 7/78 and severe 3/4<br>NICU 19/78, severe = 3/4

Separate results for severe and non severe		Mar 12, to Apr 6, 2020	Puerta de Hierro University Hospital Gregorio Marañon University 12 Octubre University Hospital,				Asthma = 6/78, severe 0/4<br>Obesity =18/78; severe = 0<br>PROM 18/78 and severe 0<br>pPROM 4/78, severe =0		**Severe COVID-19 in mothers = 4/82**<br>Maternal COVID +ve on oro-pharyngeal swab for PCR<br><br>Neonates COVID –ve = 2/82 on PCR (severe = 0)			Birthweight = 3060+3210 (Severe 1450)
Jonathan Cohen 2020 [176]<br><br>Separate results for severe and non-severe	Retros pectiv e	France	National French Survey	N=88	31 (28 to 34)	27 (4 to 40)	Smoker = 3/88<br>Diabetes=7/88	Fever = 44/88<br>Cough = 55/88<br>SOB = 40/88<br>Muscle ache = 50/88<br>Fatigue = 70/88<br>Nausea = 22/88<br>Vomiting = 14/88<br>Agueusia and/or anosmia = 67/88	History travel or meeting infected case = NR<br>**Non- Severe COVID-19 in mother = 82/88**<br>**Severe COVID-19 in mother = 6/88**<br>Maternal COVID +ve 84/88nasopharyngeal swab for PCR; 6 CT	Total deliveries = 14<br>C-section = 5<br>Vaginal = 9	Hospitalization = 17/88<br>Oxygen = 6/88	NR
Loic Sentilhes 2020 [173]	Retros pectiv e	Strasbour g, France<br><br>March 1 to April 3	Strasbourg University Hospital	N= 38	31.1 + 6.4<br><br>>35 years = 12<br><br>White = 22<br>Black 16<br>Asian = 0	Mean = 29.3 + 8.5<br>Nulliparous = 9	Obese = 4<br>Chronic hypertension = 0<br>Asthma = 1<br>Diabetes = 0<br>Chronic disease = 3<br>Tobacco = 0<br>GDM = 4<br>GH = 1<br>Preeclampsia = 2<br>IUGR = 1<br>PROM = 0	Fever = 10<br>Cough = 25<br>SOB = 13<br>Diarrhea = 7<br>Fatigue = 38<br>Sore throat = 16<br>Asonmia or ageusia = 18	History travel or meeting infected case = 38<br>**Non- Severe COVID-19 in mother = 27**<br>**Severe = 11**<br>Maternal COVID +ve 38nasopharyngeal swab for PCR	Miscarriage = 1<br>Abortion = 0<br>Termination of pregnancy = 0<br>Ongoing pregnancy = 20<br>Total delivery = 17<br>Caesarean births = 7/17<br>Because of COVID = 6/7<br>Because of pregnancy reason = 1/7<br>Vaginal = 10/17	Antiviral = 3<br>Antibiotic = 4<br>Corticosteroid = 0<br>Hydroxychloroquine = 0<br>Oxygen = 10<br>Noninvasive ventilation = 1<br>Invasive ventilation = 1<br>ECMO = 1<br>ARDS = 1<br>ICU= 3	Stillbirth= 0<br>Preterm birth = 5/17<br>Before 32 weeks = 3/17<br>Before 28 weeks = 2/17<br>Birthweight = 2810+982<br>SGA = 1/17<br>NICU admission = 3/17<br>Neonatal death = 0
Giles Kayem 2020 [177]<br><br>Separate results for severe and non-severe	Retros pectiv e	France<br><br>Mar 1 to Apr 14, 2020	33 French maternity Units	N=617	>35 = 194 (severe 18)	14-21 weeks = 105/617<br>22-31 weeks = 238/617<br>32-36 weeks = 142/617<br>>37 weeks and postpartum 132/617	Obesity = 139/617 (critical 17/35)<br>Type 1 or 2 diabetes = 14/617 (1 critical/35)<br>History of preeclampsia = 27/617 (critical 4/35)<br>Chronic hypertension = 18/617 = 3/35<br>GDM = 71/617 (critical = 3/35)<br>Smoking = 16/617 (critical 4/35)	Cough = 384/617<br>Fever = 285/617<br>Anosmia = 172/617<br>Dyspnoea = 165/617<br>Diarrhoea = 54/617	History travel or meeting infected case = NR<br>**Non- Severe COVID-19 in mother = 489/617**<br>**critical COVID-19 in mother = 35/617**<br>Maternal COVID +ve 597/617 nasopharyngeal swab for RT-PCR and 51 Chest CT<br>Neonates COVID +ve 2/190<br>**Maternal death = 1/617 (**critical **=1/35)**<br>Recovered = 486/617 (critical = 22/35)	Total deliveries = 181/617 (critical = 29/35)<br>Fetal loss (14-21 weeks) = 5/181 (critical = 0)<br>Caesarean section = 87/181 (critical 23/29)<br>Indication for c section COVID = 45/181 (critical = 22/29)	Oxygen therapy = 93/617<br>Non-invasive ventilation = 10/617<br>Intubation = 35/617<br>ECMO = 6/617	Preterm births = 50/181 (critical = 23/29)<br>Stillbirths = 7/181 (critical =2/29)<br>NICU 37/190 (critical = 13/29)<br>Neonatal death = 1/190 (critical = 1/29)
Valeria M. Savasi [178]	Cohort	Milan, Italy<br><br>Feb 23 to Mar 28, 2020	Multicentre L. Sacco (Milan), Mangiagalli (Milan), S. Gerardo MBBM Foundation (Monza), Papa Giovanni XXIII (Bergamo), and San Matteo (Pavia) as hub maternity hospitals, and Hospitals of Padua, Florence,	N= 77	32 (15 to 48)<br><br>69% Caucasian	37.29 weeks	Chronic morbidity = 24/77	Fever = 41/77<br>Cough = 50/77<br>Dyspnoea = 19/77	History travel or meeting infected case = 27 (severe 5)<br>**Non- Severe COVID-19 in mother 63**<br>**Severe COVID-19 = 14**<br>Maternal COVID +ve throat swab on PCR<br>Neonates COVID +ve = 4/56 (Severe 0) nasopharyngeal swab for PCR	Total deliveries = 57 (severe 11)<br>Caesarean delivery = 22/57 (severe 9/11)<br>Vaginal delivery = 34/57 (severe 2/11)	Antibiotic = 27/77<br>Antiviral = 25/77<br>Hydroxychloroquine = 19/77<br>Oxygen support = 20/77	Preterm birth = 12/56 (severe 4/11)<br>Birthweight = 3160 (840 to 4350) (severe 3275 (840 to 3770)<br>Apgar score at 5 min = 10 (severe 10)<br>NICU admission = 9/56 (severe 4/11)

			Lecco, Trento, Modena, Seriate and Piacenza.									
Alice Maraschini 2020 [179]	Prospective	Rome, Italy<br><br>Feb 25 to April 22	Not reported	N=47	>35 years = 12	Nulliparous = 13<br><br>Singtleton = 46<br>Multiple = 1	No comorbidity = 31<br>Comrdibity = 16<br>Obesity = 11<br>Autoimmune = 2<br>Diabetes = 2<br>Hypertension = 4<br>Smoking = 3<br>PROM = 2	Fever = 31<br>Cough = 30<br>Fatigue = 27<br>Myalgia =14<br>Sore throat = 11<br>Rhinoorhea = 6<br>Dysnpea = 15<br>Headache = 9<br>Comting/diarrhea = 9<br>Chest pain = 2<br>Conjunctivitis = 0<br>Asymptomatic = 4	**Neonatal covid +ve = 3**<br>**<24 hours =1**<br>**>24 hours = 2**	Vaginal = 24<br>Caesarean = 23<br>COVID related = 9<br>Pregnancy = 11	Antiviral = 17<br>Antbiotic = 19<br>Hydroxychloroquine = 17<br>Noninvasic eventilation = 22<br>Invasive ventilation = 9<br>ICU admission = 5<br>ECMO = 0<br>Maternal death = 0	Preterm = 15<br>Preterm < 32 = 4<br>Stillbirth = 1<br>Livebirths =47<br>LBW = 10<br>NICU admission = 13<br>Neonatal death = 0
Rebecca Pierce-Williams 2020[180]	Case Series [180]	Philidelphia, USA<br><br>March 5, April 20	12 US hospitals Multicentre	N=64	33.2±5.8<br><br>Black 28%<br>White 25%<br>Hispanic 31%<br>Asian 5%<br>Others 11%	29.9±5.8	Asthma/COPD = 16/64<br>Chronic hypertension/ cardiomyopathy = 11/64<br><br>Hypertensive disorder of pregnancy = 2/64<br>PROM = 1/64	NR	History travel or meeting infected case = NR<br>**Severe and critical COVID-19 in mothers = 44 + 20 = 64**<br>Maternal COVID +ve = 64/64 on (RT-PCR) tests on nasopharyngeal swab (63) + bronchoalveolar lavage (1)<br>Neonates COVID +ve 0<br>Maternal death = 0	Total delivered = 32<br>Caesarean birth = 24/32<br>Vaginal births = 8/ 64	Hydroxychloroquine = 52/64<br>Antibiotic = 36/64<br>Antiviral (remdesivir) = 16/64<br>Corticosteroid = 15/64 for maternal treatment<br>Anticoagulation therapy = 47/64<br>Oxygen therapy = 52/64<br>CPAP = 5/64<br>Convalescent serum = 1/64<br>Intubated = 19/64	IUGR = 2/64<br>Stillbirth = 0<br>Preterm birth = 19/32<br>Very preterm (<34) = 10/32<br>Birthweight = 2403±858 / 33<br>NICU admission = 21/33<br>Apgar score at 5 min = 7.9±1.7<br>Neonatal death = 0
Federica Di Guardo 2020 [181]	Retrospective	Italy<br><br>March 2020 to July 2020	two Italian tertiary referrals hospitals	N=145	31.5 + 5.63<br><br>Second tri =11<br>Third tri = 134	36 + 5	Respiratory disease = 111		Maternal death = 7<br><br>Neonatal covid = 7	Vaginal delivery = 108<br>C section = 37	ICU admission = 8<br>Intubated = 8	Livebirths = 145<br>Neonatal death = 10<br>Preterm birth = 55
Elias Ortiz Molina 2020 [182]	Retrpective	Alcázar de San Juan , Spain 12 march to 17 April 2020		N=20	34.9 +4.9		Obese = 2<br>Smoker = 2<br>Pre-eclampsia = 2	Fever = 14<br>Cough = 13<br>Myalgia = 7	Contact history = 7	Ongoing preg = 12<br>Vaginal birth = 3<br>C section = 5	Hydroxychloroquine = 6<br>Antibiotic = 5<br>Oxygen = 3<br>Antiviral = 3<br>Interferone = 2<br>Heparin = 16<br>ICU admission = 2	
Amily H Adhikari [183]	Observational	Texas, USA<br><br>March 18 to Aug 22, 2020	Parkland Hospital	N=252	27.0 + 6.6<br><br>Black = 18<br>White = 2<br>Hispanic 230<br>Other 2	73 Nulliparous	Mean BMI = 30.5 + 7.2<br>GDM = 14<br>Diabetes = 1<br>Hypertension = 12<br>Preeclmpaisa = 26	NR<br>Asymptomatic = 107	Mild = 132<br>Moderate = 10<br>Severe = 3	Total delivery = 239 c-section = 65 vaginal deliveries = 174		Livebirths = 251<br>Abortion = 7<br>Preterm birth < 37 = 27<br>Preterm birth < 34 = 9<br>Stillbirth = 0<br>SGA = 31
Najeh Hcini 2021 [184]	Prospective	French Guinea<br><br>June 16 to Aug 16, 2020	West French Guiana Hospital Center	N=137	25 median (21 to 31)<br>Age 35 years = 18	Nulliparous = 30	Mean BMI = 26.1 median (22.3 to 30.8)<br>Obese = 48<br>Hypertension = 2<br>CVD = 2<br>Anemia = 5<br>Preeclampsia = 15<br>GDM = 13<br>Dengue = 1<br>Hep B = 6<br>STI = 3	Asymptomatic = 87<br>Fever = 21<br>Headache = 17<br>Anosmia/ageusia = 12<br>Tiredness = 9<br>Cough = 8<br>Diarhea = 3<br>Abdominall pain = 3<br>SOB = 2	Mild to moderate = 45<br>Severe to critical = 5<br>Maternal death = 0<br><br>Covid in neonates = 0	Vaginal birth = 108<br>Caesarean birth = 19	ICU admission = 8<br>Oxygen = 3	Preterm < 37 weeks = 11<br>Preterm birth <34 weeks = 1<br>Livebirths = 127<br>Birthweight = 3090 (2755 , 3376)<br>NICU = 3
Katherine H. Campbell 2020 [185]	Prospective	Connecticut, USA April 2 to April 29	Greenwich, Bridgeport, new haven	N= 30	>35 years = 6	Nulliparous = 16		Asymptomatic = 22<br>Asymptomatic = 8	**Positive neonatal = 0**	Caesarean delivery = 10		Preterm birth = 0<br>Birthweight = 3370 + 621

William S. Vintzileos 2020 [186]	retrospective	NYC, USA<br><br>Mar 30 to April 12	NYU Winthrop Hospital	N = 32	31 years	NR	NR	Asymptomatic = 21<br>Symptomatic = 11	**NR**	NR	NR	NR
Malavika Prabhu 2020 [187]	Prospective Cohort	NY, USA<br><br>March 22 onwards	Multicentre 3 hospitals in NY<br><br>NewYork Presbyterian-Weill Cornell Medical Center, an academic tertiary care hospital, NewYork Presbyterian-Lower Manhattan Hospital, a community affiliate in Manhattan, and NewYork Presbyterian-Queens, a community teaching affiliate and tertiary care center in Queens, NY	N=70	30.9 ±3.0 (sympt)<br>31.6 ±2.3 (Asymptomatic)<br><br>White=15<br>Black = 11<br>Asian = 9<br>Hispanic = 19<br>Unknown = 35<br><br>Public insurance = 34<br>Private = 36	38.6 ± 0.71(sympt)<br>38.8 ± 0.4 (Asymptomatic)	PROM = 3/70<br>Chronic hypertension = 3/70<br>GDM = 10/70<br>Asthma = 6/70<br>Obese = 12/70<br>Smoker 1/70<br>Type 2 diabetes = 1	Asymptomatic =55/70<br>Cough = 7/70<br>Fever = 5/70	Maternal COVID +ve 70/70 nasopharyngeal swab for RT-PCR<br><br>Neonates COVID +ve 69	Total deliveries = 70<br>Caesarean births = 32/70<br>All pregnancy related<br>Vaginal deliveries = 38/70	Hydroxychloroquine = 5/70<br>Antibiotic (Azithromycin) = 2/70<br>Oxygen therapy = 3/70<br>ICU admission = 1/70<br>No treatment = 63/70	Livebirths = 69<br>Preterm birth = 11/69<br>Birthweight = 3149.6± 862.6 (Symp)<br>3060.9± 606.9 (Asympt)<br>Apgar score at 5 min = 9 ±0<br>NICU admission = 13/69<br>Fetal death = 1
Rodney McClaren 2020 [188]	Retrospective study	NY, USA	Maimonides Medical Centre	N=125	31.8 ±5.3<br><br>Severe<br>31.8±5.3	30.26 ± 4.9<br><br>Severe<br>30.9 ±3.9	PROM = 1/12<br>Obesity = 6/12<br>(Severe 32.1± 4.9)	NR	History travel or meeting infected case = NR<br>Maternal COVID +ve = 125 on oro-pharyngeal swab for PCR<br>**Severe COVID-19 in mothers = 12/125**	Discharged home with ongoing pregnancies = 3<br>Total deliveries = 9<br>Caesarean births = 7/9<br>COVID related = 6/7<br>Pregnancy complication related = 1/7<br>Vaginal delivery = 2/9	ICU admission = 9<br>Intubation = 1<br>Oxygen = 4	Preterm births = 8/9
Rasha Khoury 2020 [189]	Retrospective	NY, USA<br><br>March 13<br>April 12	Multicentre 5 USA cities<br><br>Bronx, Manhattan, and Queens (Montefiore Medical Center; Mount Sinai Health System, including Mount Sinai Hospital and Mount Sinai West; its affiliate, NYC Health and Hospitals-Elmhurst Hospital; NewYork-Presbyterian Hospital-	N=241	32 ± 3.92<br><br>Private insurance = 89<br>Government = 432<br><br>Black = 24<br>White = 67<br>Hispanic = 97<br>Others = 33	Nulliparous = 52<br>Multiparous = 149	No comorbidity = 128<br>Anemia = 16<br>Asthma = 12<br>Cardiac disease = 2<br>Dibetes = 9<br>Hypertension = 10<br>Preeclampsia = 17<br>Gestational hypertension = 15<br>GDM = 27<br>Obese = 98<br>Mean BMI30.5 ± 3.92	Asymptomatic = 148<br>Cough = 54<br>Fever = 46<br>Dyspnoea = 19<br>Myalgia = 12<br>Headache = 10<br>Anosmia = 9<br>Sore throat = 8<br>Nasal congestion = 7<br>Gastrointestinal = 6<br>Ageusia = 15<br>Others = 6	Maternal COVID +ve on oro-pharyngeal swab for PCR (RT PCR)<br><br>Maternal mortality = 0<br><br>Neonates COVID –ve = 6/245 on PCR nasophyrngeal	Total deliveries = 241<br>Vaginal deliveries = 136<br>Caesarean births = 100<br>All pregnancy related<br>Operative vaginal = 5	Maternal ICU = 17<br>Intubation = 9	Livebirths = 245<br>(233 singleton; 6 twins)<br>Preterm births < 37= 34<br>Preterm births < 34 =10<br><br>Birthweight = 3135 ±165.81<br>Mean Apgar score at 5 mins = 9± 0<br>NICU admission = 61<br>Birth asphyxia = 70/245<br>Stillbirths = 2

			Columbia University; and New York University Langone Health, including NYU Langone Hospitals, Manhattan campus, and NYU Langone Hospitals-Brooklyn									
Viktoriya London 2020 [190]	Retrospective study	NY, USA<br><br>Mar 15 to Apr 15, 2020	Brooklyn hospital	N=68	30	NR	Comorbidity = 19/68<br>Preeclampsia = 3/55	Asymptomatic = 22/68<br>Fever = 46/68<br>Cough = 46/68<br>Shortness of breath =46/68<br>sore throat = 46/68<br>Nausea/vomiting =46/68	History travel or meeting infected case = NR<br>**Non- Severe COVID-19 in mother = 68/68**<br>Maternal COVID +ve nasopharyngeal swab for PCR<br>Neonates COVID –ve = 55/55 nasopharyngeal swab for PCR<br><br>Maternal death = 0	Total deliveries = 55/ 68<br>Discharged with ongoing pregnancies = 12/68<br>Caesarean section = 22/55	Oxygen = 12/55<br>Mechanical ventilation = 1/55<br>Hydroxychloroquine = 16/55<br>Antibiotic = 16/55	Preterm birth = 9/55<br>Very preterm birth (<34) = 3/55
Erica Lokken 2020 [191]	Retrospective	Washington, USA<br><br>Jan 21 to April 17	Six hospitals in Washington	N=46	Median 29 (26-34)<br><br>Asian = 2<br>Hawaiian = 1<br>Black = 3<br>White =28<br>Multiracial; = 1<br>Other = 2<br>Unknown = 9<br><br>Hispanic = 11<br>Non-hispanic = 33<br>Unknown = 2	Second trimester = 20<br>Third trimester = 23	Type 2 diabetes = 3<br>Asthma = 4<br>Hypothyroidism = 3<br>Hypertension = 2<br>Other comorbidities = 5<br><br>Underweight = 1<br>Normal = 14<br>Overweight = 12<br>Obese = 15	Asymptomatic = 3<br>Cough = 30<br>Fever = 22<br>Congestion = 21<br>Dyspnoea = 19<br>Headache = 14<br>Loss of taste or smell = 13<br>Myalgia = 13<br>Fatigue = 12<br>Sore throat = 12<br>Other symptom = 10<br>Nausea or vomiting = 5<br>Diarrhoea = 3	History travel or meeting infected case = NR<br>Maternal COVID +ve = 46 on oro-pharyngeal swab for PCR<br>**Non-Severe COVID-19 in mothers = 40/46**<br>**Severe COVID-19 in mothers = 6/46**	Severe<br><br>Total deliveries = 8 (severe 1)<br>Discharged with ongoing pregnancy = 38 (5)<br>Caesarean section = 3 (severe =1)<br>Indication COVID related = 1/3<br>Vaginal deliveries = 5/8	Antiviral (rmdesivir)= 3<br>Hydroxychloroquine = 1<br>Corticosteroid (for mat) = 1<br>Antibiotic (ceftrizxone+azithromycin) = 1<br>ICU admission = 1	Preterm = 1/8<br>Livebirths = 7<br>Stillbirth = 1
Cassandra R. Duffy 2020 [192]<br><br>Out Universal testing	Case series	Boston, USA<br><br>April 1, 14 2020	Beth Israel Deaconess Medical Centre	N= 15	NR	NR	NR	Asymptomatic	Maternal COVID +ve on oro-pharyngeal swab for PCR (RT PCR)	NR	NR	NR
Ilona Telefus Goldfarb 2020 [193]	Prospective cohort	Boston USA<br><br>Mar 6 to May 4, 2020	Massachusetts General Hospital, Boston	N=61	29 years Hispanic and 35 non-Hispanics	24 weeks in both groups	Obesity = 26/61<br>Diabetes = 5/61<br>Asthma = 6/61	NR	History travel or meeting infected case = NR<br>**Non-severe COVI-19 in mothers = 61**<br><br>**Maternal death = 0**	NR	ICU admission =6/61<br>Intubated = 4/61	NR
Farnoosh Qadri 2020 [194]	Case series	Michigan, USA	Beaumont Hospital Dearborn	N=16<br><br>Black = 11	NR	22 to 40.3 weeks	PROM = 2/16<br>Obesity = 10/16	NR	Maternal COVID +ve on oro-pharyngeal swab for PCR (RT PCR)<br><br>Neonates COVID –ve = 10	Total deliveries = 10/16<br>Vaginal = 8/10<br>Caesarean delivery = 2<br>Pregnancy elated =2	Antibiotic (azithromycin) = 2/16<br>Hydroxychloroquine =2/16<br>Antiviral (Remdesivir) = 2/16<br>Steroid = 2/16	Preterm birth = 1/10

		March 26<br>April 10,<br>2020									Oxygen therapy = 2/16	
Ian Griffin 2020 [195]	Retrospective	NJ, USA<br><br>April 21 to May 5	two hospitals within the Atlantic Health System (AHS)	N= 27	NR	38.9 +1.5	NR	NR	Maternal COVID +ve 27 nasopharyngeal swab for PCR<br><br>Neonates COVID –ve 14/14	Total deliveries = 27<br>Vaginal deliveries= 17<br>Caesarean delivery = 10	NR	Birthweight = 3317 +501
Allie Sakowicz 2020 [196]	Retrospective	Chicago, USA<br><br>April 8 to May 31.	North-western Memorial Hospital	N=101	30.6 +/- 18.2<br><br>Public insurance = 62<br><br>Aisan =3<br>Black = 28<br>White = 23<br>Other 46<br>Latin = 53	Nulliparous = 30	Tobacco use = 9<br>Diabetes = 1<br>Hypertension = 7<br>Pulmonary disease = 22<br>GDM = 6<br><br>Obese = 35<br>Mean BMI = 32.3 +3.4	Asymptomatic = 24<br>Symptomatic = 77				
Dilek Sahin 2020 [197]	Prospective study	Ankara, Turkey<br><br>March 11 to Sep 10, 2020	Ministry of Health Ankara City Hospital	N=533	Age 28.04+5.84	24.97 + 11.16<br>First tri = 130<br>Second = 15<br>Third = 238	Mean BMI 26.49+ 5.47<br>Obese = 71<br>Hypothyroidism = 28<br>Hypertnsion = 23<br>Diabetes = 8<br>Asthma = 8<br>Rhtutomologcal disease = 7<br>Cardiovasuclar = 5<br>Thalesemia = 2<br>Diabetes 1 = 3<br>Renal disease = 2<br>Epilesy = 2<br>ITP =<br><br>IUCG = 5<br>GDM = 3<br>GH = 4<br>Pre-elcmpasia = 5<br>Placental abruption = 1<br>DVT = 1	Asymptomatic = 165<br>Cough = 178<br>Myalgia = 168<br>Dyspnea = 99<br>Sore throat = 83<br>Fever = 71<br>Headache = 65<br>Anosmia = 64<br>aGeusia = 46<br>nausea/vomiting = 32<br>nasal congestion = 28<br>diarrhea = 21<br>chest pain = 12	Close contact = 128/533<br>**Non-severe = 516/533**<br>**Severe = 17 / 533**<br>Maternal COVID +ve on oro-pharyngeal swab for PCR (RT PCR)<br><br>Neonatal COVID positive = 0<br><br>Materna; mortality = 2	Delivered = 131<br>Ongoing prey = 389<br>Vaginal delivery = 44<br>Cesarean = 87	Anticoagulant = 220<br>Hydroxychloroquine = 55<br>Antiviral (Lipinavir.ritonir) = 39<br>Corticosteroid = 20<br>Antibiotic (Azithromycin) = 17<br>Plasma = 3<br>Immunotherapy = 1<br><br>Oxygen = 17<br>Invasic mechanical ventilation = 2<br>ICU admission = 7	Preterm delivery = 22<br>Pregnancy loss = 13<br>Livebirths = 132 (128 singleton; 2 twins)<br>Birthweight = 3100.80 + 626.62<br>Apgar score at 1 min = 8<br>Apgar score at 5 min = 9<br>NICU admission = 13/132
Mehmet Yekta Oncel 2020 [198]	Cohort	Istanbul, Turkey	Multicentre cohort	N=125	Urban = 119<br>Rural = 6	36.7 + 0.5<br>34.2 + 4.2	GDM = 7/125<br>Preeclampsia = 6/125<br>Hypertension = 2/125<br>Placenta previa = 1 /125<br>Smoking =8/125	NR	**Health care workers = 8**<br><br>**Non- Severe COVID-19 in mother = 117**<br>**Severe = 8**<br>Maternal COVID +ve nasopharyngeal swab for PCR<br><br>Neonates COVID +ve 4 on nasopharyngeal swab RNA<br><br>Maternal deaths = 6	Total deliveries = 125<br>Caesarean births = 89<br>Vaginal deliveries = 36	Mechanical ventilation = 8/125<br>ICU admission = 8	Livebirths = 125<br>NICU admission = 34<br>Preterm births (<37 weeks) = 33/125<br>Birthweight = 3117.5 + 124.7 (COVID-19 –ve)<br>2437.5 + 886.4<br>LBW = 16/125<br>Apgar score at 1 min 7.7 + 0.19; 7.2 + 1.4<br>Apgar score at 5 min = 9.9 + 0.19; 8 + 0.9<br>Neonatal mortality = 1
Jie Yan 2020 [199]	Case series	Hubei province, China<br><br>Jan 20 to Mar 24, 2020	Multicentre 25 hospitals in china	N=116	NR	38 weeks	GDM = 9 (7.8)<br>Hypertension = 5 (4.3)<br>Preeclampsia = 4 (3.4)	Fever = 59 (50.9%)<br>Cough = 33 (28.4%)<br>Fatigue = 15 (12.9)<br>SOB = 9 (7.8)<br>Sore throat = 10 (8.6)<br>Myalgia = 6 (5.2)<br>Dyspnoea = 3 (2.6)<br>diarrhoea = 1 (0.9)<br>Asymptomatic = 27 (23.3%)	History travel or meeting infected case = NR<br>**Non-severe COVID-19 in mothers = 108**<br>**Severe COVID-19 in mother = 8**<br>Maternal COVID +ve = 116 (100%) on throat swab on PCR + CT scan<br>Maternal death = 0	Total deliveries = 99<br>Caesarean births = 85 (85.9%)<br>COVID related 33 (38.8)<br>Pregnancy related = 52 (62.2%)<br>Vaginal deliveries = 14 (14.1%)<br>Abortion = 1	Antibiotic (Piperacilin, sulbactam, Imipenam, Moxifloxacin, Cephalosporin, linezolid, meropenam, polymexicin, sulphanilamide, azithromycin, levofloxacin, vancomycin, omidazole) = 109<br>Antiviral (Oseltamivir, Ganciclovir, Arbidole,	Preterm birth 21/99 (21.2%)<br>PROM = 6/99<br>Neonatal asphyxia = 1<br>Birthweight = 3108 +/- 526<br>Apgar score = 9<br>Apgar score at 5 min = 10<br>NICU admission = 47 (47%)<br>Neonatal death = 1

											interferon, lopinavir, peramivir, ribavirin) = 63<br>Corticosteroid (methylprednisolone) (for mat) = 37<br>ICU admission = 8<br>Non-invasive ventilation = 6<br>Invasive ventilation = 2<br>ECMO = 1<br>Plasmapheresis = 1	
Zhang Lu 2020 [200]<br><br>Chinese study	Case series	Wuhan, China<br><br>Jan 30 to Feb 17, 2020	Wuhan University People's Hospital	N = 16	29.3+2.9	38.7+1.4	GDM = 3<br>Pre-eclampsia = 1	Cough = 16<br>Diarrhoea = 16	History of travel or contact with +ve case = NR<br>**Non-Severe COVID-19 in mothers = 15**<br>**Severe COVID-19 in mothers = 1**<br>Maternal COVID +ve = 16 (100%)<br>Neonatal COVID +ve = 0 out of 10 analysed on pharyngeal swab	Total number of deliveries = 16<br>Caesarean sections = 16 (100%)	NR	PROM = 3 (18.75%)<br>Fetal distress = 1 (6.25%)<br>Placental abruption = 1<br>Livebirths = 16<br>Mean Birthweight = 3139+437<br>Preterm birth = 3 (18.75%)<br>Neonatal asphyxia = 1 (6.25%)<br>Neonatal death = 0
Lian Chen 2020 [201]<br><br>Given as severe and non-severe separately	Case series	Wuhan, China<br><br>\Dec 8, 2019 to March 20, 2019	Wuhan	N=118	Median 31 (28-34)	NR	NR	Asymptomatic = 6<br>Fever = 84 (75%)<br>Cough = 82 (73%)<br>Chest tightness = 20 (18%)<br>Fatigue = 19 (17%)<br>Dyspnoea = 8 (7%)<br>Diarrhoea = 8 (7%)<br>Headache = 7 (6%)	History of travel or contact with +ve case = 25 yes; 1 NR; 92 No<br>**Non-Severe COVID-19 in mothers = 109**<br>**Severe COVID-19 in mothers = 9**<br>Maternal COVID +ve = 118 (100%)<br>84 Laboratory diagnosed + 34 clinically diagnosed<br>Neonatal COVID +ve = 0	Total number of deliveries = 68 (58%)<br>Caesarean section = 63 (93%)<br>Vaginal deliveries = 5 (7%)<br>Discharged with ongoing pregnancy = 41 (35%)<br>Abortion = 9 (8%)	NR	Livebirths = 70 (100%)<br>Preterm birth = 14 (21%)<br>Median Apgar score at 1 min = 9 (8-9) * out of 66<br>Neonatal asphyxia = 0<br>Neonatal death = 0
Yangli Liu 2020 [202]	Case series	Wuhan, China<br>Dec 8, 2019 to Feb 25, 2020	Hospital of Sun Yat-sen University	N = 13	Range 22 to 36		PROM = 1<br>Fetal distress = 3	Fever = 10 (76.9%)<br>Dyspnoea = 3 (23.1%)	Travel history to Wuhan or close contact to positive case = 13 (100%)<br>**Non- Severe COVID-19 in mother=12**<br>**Severe COVID-19 = 1**<br>Maternal COVID +ve = 13 (100%)<br>Laboratory confirmed<br>Neonates COVID +ve = 0	Total number of deliveries = 10<br>Caesarean sections = 10 (100%)<br>Reasons for C-section = fetal distress =3 (30%)<br>PROM = 1 (10%)<br>Discharged with ongoing pregnancy = 3	Oxygen = 1<br>ICU = 1<br>Intubate =1<br>MODS = 1<br>ARDS = 1<br>ECMO= 1	Stillbirth = 1 (10%)<br>Preterm birth = 6 (60%)<br>Livebirths = 9 (90%)
Jing Liao 2020 [203]<br><br>Individual cases given	Retros pectiv e	Wuhan China<br><br>Jan 20 to Mar 2, 2020	Zhongnan Hospital	N=10	31.9 + 3.34	38.7 + 1.42 weeks	NR	Fever =5<br>Cough =3<br>Sore throat =1<br>Dyspnoea = 0<br>Chest pain = 0<br>Myalgia =0<br>Diarrhoea = 0	History travel or meeting infected case = NR<br>**Non- Severe COVID-19 in mother**<br>Maternal COVID +ve =10 on throat swab test + Chest X ray<br>Neonates COVID –ve = all nasopharyngeal swab for PCR	Total deliveries = 10<br>Vaginal delivery = 10	NR	Total live births =10<br>Preterm birth =1<br>Neonatal asphyxia = 0<br>Birthweight 3283g + 449
Xu Qiancheng 2020 [204]	retros pectiv e	Wuhan China<br><br>Jan 15 to Mar 15, 2020	Yijishan Hospital of Wannan Medical College	N=28	30 +1.28	38 +0.61<br><br>First trimeter = 3<br>Second trimester = 1<br>Third trimester = 24	Hypertension = 1/28<br>Diabetes = 2/28<br>Hepatitis = 2/28<br>Hypothyroidism = 1/28	Fever = 5/28<br>Malaise = 1/28<br>Cough = 7/28<br>Dysnpnea = 2/28<br>Abdominal pain = 5/28	**Non- Severe COVID-19 in mother= 26**<br>**Severe = 2**<br><br>Neonates COVID –ve = 0<br>Maternal death = 0	Caesarean section = 17<br>Vaginal = 5<br>Medical abortion = 4<br>Ongoing pregnancies = 2	Antiviral (ribavirin, Umifenovir, and interferone) = 21/28<br>Antibiotics (cephalosporin, quinolone) = 24<br>Corticosteroid = 4<br>Immunoglobulin = 3<br><br>Hopsitalization = 7/28	Livebirths = 23 (singleton = 21, 1 twin)<br>Preterm birth = 1<br>Birthweight = 3130+ 117.11<br>LBW = 1<br>Apgar score at 1 min = 10 +0<br>Apgar score at 5 min 10 +0<br>Neonatal asphyxia = 0<br>NICU admission = 0
Fang Liu 2020 [205]	Retros pectiv e	Shangai, China<br><br>Jan 23 to March 4	Shanghai Children's Medical Center	N=21	31+1	NR	NR	Fever = 8<br>Cough = 6<br>SOB = 1<br>Fatigue 8<br>Loss of appetite = 2	**Exposure = 8**	NR	ICU admission = 1<br>Mechanical ventilation = 1	NR
Rui Nei 2020 [206]	Retros pectiv e	Hubei, China<br>Jan 1 to Feb 20, 2020	Yichang Central People's Hospital	N= 33	30.5 + 3.1	8 Nullipaorus =<br>25 Multiparous	Cardiovascular = 9<br>Depressive =<br>Digestive = 1<br>Endocrine = 2<br>Nervous system = 1<br>Infectious disease = 1<br>PPROM = 3	Asymptomatic = 4<br>Fever= 21<br>Cough = 13<br>Fatigue = 7<br>SOB = 7<br>Diarrhea = 6<br>Myalgia = 5<br>Sore throat = 5	**Travel/contact history = 32; 1 no**<br><br>**Non- Severe COVID-19 in mother= 32**<br>**Severe = 1**<br><br>Neonates COVID –ve = 1	Total deliveries = 27<br>Ongoing pregnancies = 5<br>Induced abortion = 1<br>Vaginal deliveries = 5<br>Caesarean section = 22	Oxygen therapy = 30<br>Non-invasive ventilation = 1<br>Invasive ventilation = 0<br>Antiviral = 33<br>Antibiotic = 29<br>Corticosteroid = 11<br>Chinese medicine = 12<br>ICU admission = 0	Livebirths = 28 (singleton 26, 1 twin)<br>Preterm birth = 10/28<br>Birthweight = 2988+ 502<br>LBW = 5<br>Apgar score at 1 min = 8.9 + 0.8<br>Apgar score at 5 min = 9.7 + 0.5<br>NICU admission = 1<br>Neonatal death = 0

							Gestational hypertension = 2<br>GDM = 2<br>Fetal distress = 4	Chest pain = 1<br>Abdominal pain = 1	**Maternal death = 0**		ARDS = 1	
Aya Mohr-Sasson 2020 [207]	Case series	Israel<br><br>March to April 2020	university-affiliated tertiary medical centre	N=11	Age = 33.2 +9.1	36.6+ 1.03<br><br>Third trimester = 11/11		Fever = 3/11<br>Weakness = 5/11<br>Respiratory = 6/11<br>Gastrointestinal = 2/11<br>Others = 2/11	Non-severe = 10/11<br>Severe = 1/11<br>Maternal COVID +ve on oro-pharyngeal swab for PCR (RT PCR) 11/11	Deliveries = 11/11<br>Ongoing pregnancies = 6/11<br>Caesarean birth = 2/11 and 2 related to COVID related<br>Vaginal deliveries = 9/11	Hospitalised = 7/11<br>Intubated = 1/11	NR
Arun Harishchandra Nayak 2020 [208]	Cohort	Mumbai, India<br><br>Apr 1 to May 15, 2020	Medical College, central Mumbai	N= 141	< 20 = 8<br>21-25 = 64<br>26-30 = 57<br>>30 12	Nulliparous = 55<br>Multiparous = 86<br><br>All third trimester	Diabetes = 4<br>GDM = 4<br>Hypertension = 7<br>Pre-eclampsia = 7<br>Asthma = 24hypothyroidism = 5<br>Anaemia = 5<br>HIV = 1<br>Others = 2<br>Total = 27		Maternal COVID +ve on oro-pharyngeal swab for PCR (RT PCR<br><br>**Maternal deaths = 3**<br><br>Neonates COVID –ve =3	Total deliveries = 134<br>Vaginal deliveries = 66<br>Instrumental = 1<br>Caesarean section = 67<br>Abortion = 6<br>Ectopic pregnancy = 1		LBW = 39/131<br>Stillbirths = 3<br>NICU admission = 24
Ceulemans 2020 [209]<br><br>Universal screening	Cohort	Belgium	North-East Flanders	N=13				Asymptomatic = 8<br>Upper airway symptoms = 4	**Non- Severe COVID-19 in mother = 13**<br><br>**Maternal death = 0**		ICU admission = 0	
Jayasree Santhosh 2020 [210]<br><br>Non-severe and severe	Retrospective	Oman	Royal Hospital	N=60	32 + 6	Median 35 (30 to 37)	Hypertension = 3<br>Diabetes = 5<br>GDM = 15<br>Haematological disease = 8<br>Anaemia = 26<br>PROM = 1<br>Pre-eclampsia = 1	Asymptomatic = 16<br>Fever = 18<br>Cough = 24<br>Rhinorrhoea/sore throat = 13<br>Myalgia = 8<br>Fatigue = 1<br>Headache = 9<br>SOB = 8<br>Vomiting/diarrhea = 12<br>Anosmia/ageusia = 5	**Non- Severe COVID-19 in mother = 50**<br>**Severe COVID-19 in mother = 10**	Deliveries = 48<br>Ongoing pregnancy = 8<br>Vaginal delivery = 24<br>Instrumental delivery = 4<br>Elective 13aesarean = 6<br>Emergency caesarean = 14<br>COVID related caesarean = 3<br>Obstetric related = 22		Preterm birth (34 to 37) = 14<br>Preterm birth (24 to 34) = 4<br>Miscarriage = 4<br>Stillbirth = 1/47<br>LBW < 2500 = 15/ 47
Muhammad Azeeem Khan 2020 [211]	Retrospective	Karachi, Pakistan<br><br>27 April to 16 June 2020	Indus Hospital	N=66	-	-	-	-	**Non- Severe COVID-19 in mother = 66**<br><br>**Neonates = all negative**	Delivered = 66<br>Vaginal delivery = 20<br>Caesarean births = 47 (elective =7; emergency = 40)	-	Live births = 67 (65 singleton and 1 twin)
Brisandy Ruiz Mercedes 2020 [212]	Retrospective	Dominican Republic<br><br>March 20 June 30, 2020	Single centre	N= 154	29.87 + 5.83	32.31 + 3.68	BMI = 28.0 + 2.6	SOB = 10/15<br>2 = palpitations<br>fatigue = 1	**All moderate disease = 154**<br>**Severe COVID = 34**<br><br>**Matrna; death = 2/15**	Delivered = 15/15<br>Ceasarean = 15/15	ICU admission = 34<br>Intubate = 13	Preterm delivery = 9/15<br>Neonatal death = 1/154<br>Birthweight = 2200 + 700<br>LBW = 7/15<br>NICU admission = 5/15
Mahtab Sattari 2020 [213]	Retrospective	Hamadan - Iran<br><br>Jan 6 to June 21, 2020	hospitals of Hamadan Province	N=50	29.20 + 5.8 (18-38)	28.42 + 8.2	Smoking = 3<br>Diabetes = 1<br>Hypertension = 2<br>Heart disease = 2	Fever = 30<br>Cough = 27<br>Myalgia= 9<br>Fatigue = 6<br>Diarhhea = 2<br>Nausea = 9<br>Duspnea = 27	**Recent travel = 20**<br><br>**Non-severe = 42**<br>**Severe = 8**<br><br>**Maternal death = 2**<br><br>**Newborn Positive = 7**	Ongoing pregnancy = 26<br>Deliveries = 24<br>Vaginal delivery = 14<br>Caesarean birth = 10	ICU admission = 4	Preterm birth = 7<br>Apgar score at 1 min = 8.12 + 1.61<br>Apgar score at 5 min = 9.24 + 1.2
Ensiyeh Jenabi 2020 [214]	Case-control	Hamadan - Iran<br><br>Sep 1 to Nov 15, 2020	Hamadan University of Medical Sciences	N=90	29.47+5.64 and 28.78 + 6.92	31.13 + 2.87 and 37.26 + 2.56	Any comorbidity = 22<br>Pre-eclampsia = 15	Asymptomatic = 45	**Ytavel history = 14**	c-section = 39	Hospitalization = 2	Peterm birth = 18<br>LBW = 16<br>Neonatal death = 3

Soudabeh Kazemi Aski [215]	Retrospective	Rasht, Iran<br><br>Early Mar to Late Apr 2020	Al Zahra Hospital	N=70			<25 BMI = 15<br>26-29 18<br>>29 = 28<br>Diabetes = 5<br>Hypothyroidism = 11<br>Hypertension = 2<br>Renal disease = 2<br>Migraine = 1	Fever = 54<br>Cough = 27<br>Myalgia = 20<br>SOB = 16<br>Chills =13<br>Sore throat =7<br>Runny nose = 5<br>Headache = 5<br>Sickeness = 3<br>Anorexia = 3<br>Order disorder = 2<br>Taste disorder = 2<br>Diarrhea = 1	**14 close contact history**<br><br>**Maternal deaths = 2**	C section = 24<br>Vaginal births = 8	Antibiotics = 57<br>Corticosteroid = 5<br>Antiviral = 50<br>ICU admission = 2	Livebirths - = 34<br>Singleton = 32, twin = 1<br>peterm births = 15<br>NICU admission = 4<br>Neonatal deaths = 2
Citra NZ Mattar 2020 [216]	Propective study	Singapore<br><br>15 Mar to 22 Aug 2020	4 tertiary hospitals	N=16	Age range 23-36<br><br>Asia = 13<br>White = 3	First trimester = 6<br>Second trimester = 7<br>Third trimester = 3	Asthma = 2	Asymptomatic = 3<br>Rhinorrhea = 5<br>Anosmia/ageusia = 4<br>Sorethroat = 9<br>Cough = 9<br>Diarhea = 2<br>Fever = 4	**Contact and travel history = 9**	Ongoing preg = 9<br>Miscarriage = 2<br>Vaginal = 5	Oxygen = 1	
Katarina Remaeus 2020 [217]	Retrospective	Stockloh m Sweden<br><br>March 19 to April 26, 2020	Karolinska Institute	N=67	32 (19-42)		BMI = 27 (18-38)<br>Underweight = 25<br>Overweight = 22<br>Obese = 19<br>Smoking = 66<br>Preeclmapsia = 14<br>Asthma = 6<br>Diabetes = 10			Total deliveres = 67<br>Vaginal = 47<br>Caesarean = 20	ICU admission = 4	Livebirths = 67 (singleton = 66; 1 Twin)<br>Preterm birth = 13<br>Pretem 32-37 = 9<br><32 = 3<br><28 = 2<br>Stillbirths = 1<br>Neonatal death = 1<br>SGA = 3<br>NICU admission = 12
Amihai Rottenstreich 2020 [218]	Retrospective	Israel<br><br>March 15 to July 4	7 uni hospitals	N=52	28 (24-35)<br>>35 = 16<br>Nulliparous = 9		GDM = 6<br>GH = 7	Fever = 11<br>Cough = 14<br>Dyspnea = 6<br>Sorethroat = 4<br>Gastrointestinal 4<br>Anosmia/aguesua = 5		Vaginal deliveries = 39<br>Caesarean = 13	Intubation = 1<br>ICU admission = 1	Preterm birth = 17<br>< 34 weeks = 9<br>Birthweight = 3280 (2927 – 3606)<br>NICU amdmission = 6
Mohsen A A Farghaly 2020 [219]	Retrospective	New Yok, USA<br><br>March to May 2020	Brookedale University Hospital	N=15	33.40 + 7.2<br>Black = 11<br>Hispanics = 2<br>Others = 2		Smoker = 0<br>Alcohol = 0<br>Comorbidities = 6	Fever = 1<br>Sorethroat = 3<br>Cough = 4<br>Dyspnea = 2<br>Hypoxia = 0<br>Myalgia= 3<br>Diarrghea = 1<br>Asymptomatic = 9	**Neonatal positive = 3**	Delivered =15<br>Vaginal 15<br>Caesarean = 10		Apgar score at 1 = 7.4 + 2.5<br>Apgar score at 5 mins = 8.33 + 1.4<br>Preterm = 2<br>Birthweight = 2800 + 100<br>NICU admission = 10
Laura D Zambrano 2020 [220]	Retropespctive	USA<br><br>Jan 22 to Oct 3, 2020	National data	N= 23434	<35 = 19414<br>>35 = 4020<br>Hispanic = 6962<br>Asian = 560<br>Black = 3387<br>White = 5508<br>Others = 958		Diabete= 427<br>CVD = 304<br>Lund dis= 506<br>Kidney dis = 18<br>Liver dis = 17<br>Immunocompromised = 124<br>Newurological problems = 44<br>Psychiatric = 62<br>Autoimmune = 26<br>Severe obesity = 174<br>Any comorbidity = 7795	Cough = 5230<br>Fever = 3328<br>Myalgia = 3818<br>Chills = 2537<br>Headache = 4447<br>SOB = 2692<br>Sorethroat = 2955<br>Diarrhea 1479<br>Nausea/vomiting = 2052<br>Abdominal pain = 870<br>Runny nose = 1328<br>Anosmia/ageusia = 2234<br>Fatigue = 1404<br>Wheezing = 172<br>Chestpain = 369	**Maternal death = 34**		ICU admission = 245<br>ECMO = 174	
N la Cour Freiesleben 2020 [221]	Cohort	Denmark<br><br>Feb 17, April 23, 2020	Danish Detal Medicine database	N=18	-	-	-	-	**-**	Ongoing pregnancy = 17<br>Pregnancy loss = 1	-	-

Monia Rios-Silva 2020 [222]	Retrospective	Mexico<br><br>Until May 25, 2020	Ministryt of health	N=448	Median = 29 (25-33)		Diabetes = 17<br>COPD = 1<br>Asthma = 11<br>Immunosuprression = 7<br>Hypertension = 17<br>Obesity = 59<br>CVD = 2<br>Kidney disease = 2<br>Smoking = 12<br>Another comorbidities= 32		**Maternal death = 10**		Mechanical ventilation = 7<br>ICU admission = 14	
Roman G. Shmakov 2020 [223]	Prospective	Moscow, Russia	Research was carried out at the National Medical Research Center for Obstetrics, Gynecology and Perinatology, Ministry of Healthcare	N=66	30.3 ± 6.25	31.3 ± 10.4<br><br>1st trimester = 5<br>2nd trimester = 7<br>3rd trimester = 54	Mean BMI = 27.1 ± 4.6<br>Anemia = 11	Cough = 34<br>Pharyngitis = 8<br>Fever = 22<br>Anomia = 23<br>SOB = 11<br>Fatigue = 11<br>Asymptomatic = 15	**Non-severe COVID-19 = 60**<br>**Severe COVID-19 = 6**<br><br>**Neonatal positive for COVID-19 = 0**<br><br>**Maternal death = 1**	Abortion (<20 weeks) = 4<br>Ongoing pregnancy = 20<br>Delivered = 42<br>Caesarean delivery = 17<br>Vaginal deliveries = 23<br>Vacuum extraction = 2	Anticoagulant (low molecular weight heparin) = 66<br>Interferon alfa-b = 66<br>Antimicrobial (amoxicillin/clavulanic acid) 32<br>Lopnavir/Ritonavir = 6<br>Oxygen therapy = 6<br>CPAP = 6<br>Mechanical ventilation = 2<br>Corticosteroid (dexamethasone) = 6<br>Immunoglobulin = 6	Births = 42<br>Preterm birth = 8<br>Mean birthweight = 3283 + 477<br>Apgar score at 1 min = 7.8 + 0.6<br>Apgar score at 5 min = 8.7 + 0.5<br>Perinatal mortality = 0
Amal Ayed 2020 [224]	Retrospective	Kuwait<br>Mar 2020 to May 31, 2020	Nationwide	N=185	31 median (27.5 to 34)	Nulliparous = 36<br><br>1st tri = 21<br>2nd tri = 64<br>3rd tri = 95	Hypothyroidism = 7<br>Asthma = 4'diabetes = 1artritis = 1<br>Anxiety = 1<br>Peptic ulcer = 1<br>Crohns disease= 1<br>Thalassemia = 1<br>GDM = 19<br>Pre-eclampsia = 4<br>Fetal distress = 9	Fever = 105<br>Cough = 90<br>Anosmia = 10<br>Sorethroat/rhinorrhoea = 42<br>Malaise/fatigue = 26<br>Chest pain/SOB = 22<br>Vomiting/diarrhoea = 11<br>Asymptomatic = 21	**COVID positive neonate = 0**	Ongoing pregnancy = 16<br>Miscarriage = 3<br>c-section = 79<br>vaginal delivery = 86	Antiviral = 182<br>Antibiotic = 50<br>Heparin = 179<br>ICU admission = 2	Livebirths = 165<br>Birthweight = median 3006 (2750 to 3215)<br>Mean apgar at 1 min = 8+1<br>Apgar score at 5 min = 9 + 1

Supplementary File 5a: Methodological quality of Cohort

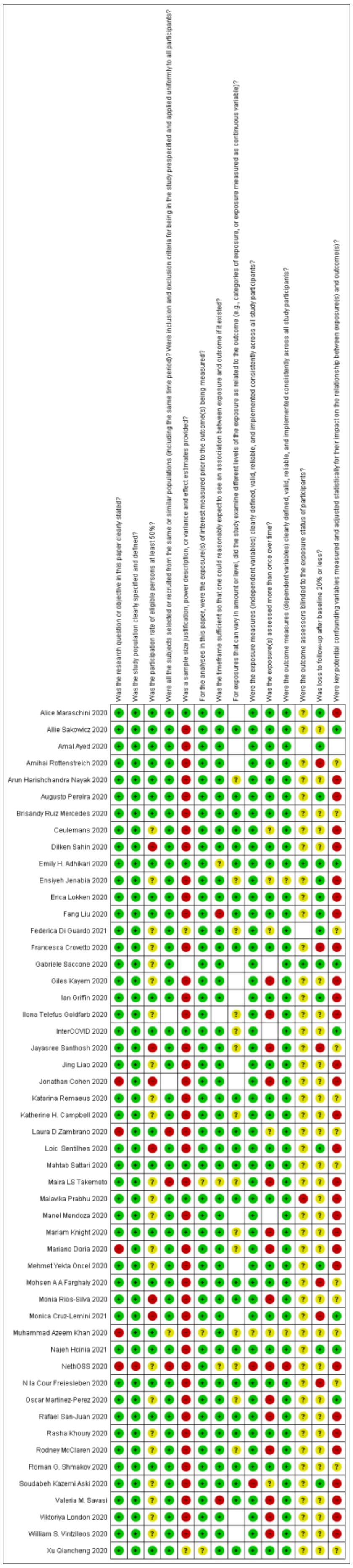

Supplementary File 5b: Methadological quality of Case Series

	Was the study question or objective clearly stated?	Was the study population clearly and fully described, including a case definition?	Were the cases consecutive?	Were the subjects comparable?	Was the intervention clearly described?	Were the outcome measures clearly defined, valid, reliable, and implemented consistently across all study participants	Was the length of follow-up adequate?	Were the statistical methods well-described?	Were the results well-described?
Aya Mohr-Sasson 2020	+	+	+	+	+	+	+	+	+
Cassandra R. Duffy 2020	+	+	+	+	+	+	?	−	+
Citra NZ Mattar 2020	+	+	+	+	+	+	+	+	+
Elias Ortiz Molina 2020	+	−	−	+	+	+	+	+	+
Farnoosh Qadri 2020	+	+	+	+	+	+	+	−	+
Jie Yan 2020	+	+	+	+	+	+	+	+	+
Lian Chen 2020	+	+	+	+	+	+	?	−	+
Rebecca Pierce-Williams 2020	+	+	+	−	+	+	+	+	+
Rui Nei 2020	+	+	+	+	+	+	+	+	+
Yangli Liu 2020	+	+	+	+	+	+	+	−	+
Zhang Lu 2020	+	+	+	+	+	+	+	+	+

References

1. Kolkova Z, Bjurstrom M, Lansberg J, Svedes E, Hamer MA, Hansson SR, et al. Obstetrics and intensive-care strategies in a high-risk pregnancy with critical respiratory failure due to COVID-19: a case report. Case Reports in Women's Health. 2020;27:e00240.
2. Abourrida Y, Rebahi H, Oussayeh I, Chichou H, Fakhir B, Soummani A, et al. Case Report: management of severe COVID-19 in pregnancy. Case Reports in Obstetrics and Gynecology. 2020;2020:8852816.
3. Slyton-Milam S, Sheffels S, Chan D, Alkinj B. Case Report: induction of labor in an intubated pateint with coronavirus disease 2019 (COVID-19). Obstet Gynecol. 2020:1-3.
4. Cheng SO, Khan S, Z A. Maternal death in pregnancy due to COVID-19. Ultrasound Obstet Gynecol. 2020;56:117-24.
5. Forero-Pena D, Rodriguez MI, Flora-Noda DM, Maricuto AL, Velasquez VL, Soto LM, et al. The first pregnancy woman with COVID-19 in Vanezuela: pre-symptomatic transmission. Travel Med Infect Dis. 2020;36:101805.
6. Polonia-Valente R, Moucho M, Tavaresa M, Vilan A, Montenegron N, Rodrigues T. Vaginal delivery in a women infected with SARS-CoV-2 - the first case reported in Portugal. Eur J Obstet Gynecol Biol. 2020;250:253-54.
7. Polinx B, Kieffer D, Michiels I, Petermans S, Strybol D, Delvaux S, et al. Vertical transmission of SARS-CoV-2 infection and pretrm birth. Eur J Obstet Gynecol Biol. 2020;https://doi.org/10.1007/s10096-020-03964-y.
8. Easterlin MC, Beritto TD, Yeh AM, Wertheimer FB, Ramanathan R. Estremely preterm infant born to a bother with severe COVID-19 pnuemonai Journal of Investigateive Medicine High Impact Case Reports 2020;8:1-5.
9. Ferrailo A, Barra F, Kratochwila C, Paudice M, Vellone VG, Godano E, et al. Report of positive placental swabs for SARS-CoV-2 in an asymptomatic pregnanct woman with COVID-19. Medicina. 2020;56:306.
10. Yu Y, Fan C, Bian J, Shen Y. Severe COVID-19 in a pregnant pateint admistted to hospital in Wuhan. Int J Gynecol Obstet. 2020;First published: 19 May 2020 https://doi.org/10.1002/ijgo.13232.
11. Mehta H, Ivonovic S, Cronin A, VanBrunt L, Mistry N, Miller R, et al. Novel coronavirus-related acute respiratory distress syndrome in a patient with twin pregnancy: A case report. Case Reports in Women's Health. 2020;27:e00220.
12. Ahmed I, Azhar A, Eltaweel N, Tan BK. First Covid-19 maternal mortality in the UK associated with thrombotic complications Bri J Hematol. 2020;190:e1-38.
13. Kuhurt K, McMicking J. Placental abruption in a twin pregnancy at 32 weeks' gestation complicated by coronavirus disease 2019 without vertical transmission to the babies. Am J Obstet Gynecol MFM. 2020;doi: 10.1016/j.ajogmf.2020.100135 [Epub ahead of print].
14. De Socio GV, Malincarne L, Arena S, Troiani S, Benedetti S, Camilloni B, et al. Delivery in asymptomatic Italian woman with SARD-CoV-2 infection Mediterr J Hematol Infect Dis. 2020;12(1):e2020033.
15. Martinelli I, Ferrazzi E, Ciavarella A, Erra R, Iurlaro E, Ossola M, et al. Pulomoary embolism in a young pregnant woman with COViD-19. Thrombosis Research. 2020;191:36-7.
16. Vallejo V, Ilagan J. A postoartum death due to coronavirus disease 2019 (COVID-19) in the United States Osbetr Gylnecol. 2020;136(1):52-5.
17. Blauvelt C, Chiu C, Donovan A, Prahl M, Shimotake T, George R, et al. Acute respiratory distress syndrome in a preterm pregnant patient with coronavirus disease 2019 (COVID-19). Obstet Gynecol. 2020;136(1):46-51.
18. Hong L, Smith N, Keerthy M, Lee-Griffith M, Garcia R, Shaman M, et al. Severe Covid-19 infection in pregnancy requiring intubation without preterm delivery: a case report. Case Reports in Women's Health. 2020;27:e00217.
19. Lu D, Sang L, Du S, Li T, Chang Y, Yang X. Asymptomatic COVID-19 infection in late pregnancy indicated no vertical transmission. J Virol. 2020;First published: 24 April 2020 https://doi.org/10.1002/jmv.25927.
20. Lyra J, Valente R, Rosario M, Guimaraes M. Cesarean section in a pregnant woman with COVID-19: first case in Portugal. Acta Med Port. 2020;33(6):429-31.
21. Baud D, Greub G, Favre G, Gengler C, Jaton K, Dubruc E, et al. Second trimester miscarriage in a pregnant woman with SARS-CoV-2 infection. JAMA. 2020;323(21):2198-200.

22. Li J, Wang Y, Zeng Y, Song T, Pan X, Jia M, et al. Critically ill pregnant patient with COVID-19 and neonatal death within two hours of birth. Int J Gynecol Obstet. 2020;First published online: 27 May 2020: DOI: 10.1002/ijgo.13189.
23. Romero DG, Perez JO, Bautista LG, Santana-Cabrera L. Pregnancy and perinatal outcome of a woman with COVID-19 infection. Revista Clinica Espanola. 2020;doi: 10.1016/j.rceng.2020.04.005 [Epub ahead of print].
24. Xia H, Zhao S, Wu Z, Luo H, Zhou C, Chen X. Emergency caesarean delivery in a patient with confirmed COVID-19 under spinal anaesthesia. Br J Anaesth. 2020;124(5):e216-18.
25. Browne PC, Linfert JB, Perez-Jorge E. Successful treatment of preterm labour in association with COVID-19 infection. Am J Perinatol. 2020;37(8):866-68.
26. He S, Wang D, Ruibin C, Ding D, Yu Y, He M, et al. Death of a neonate born to a critically ill mother with COVID-19: a case report. Chin J Perinatal Med. 2020;23(4):217-20.
27. Kalafat E, Yaprak E, Cinar G, Varli B, Ozisik S, Uzan C, et al. Lung ultrasound and computed tomographic findings in pregnant woman with COVID-19. Ultrasound Obstet Gynecol. 2020;First published: 06 April 2020 https://doi.org/10.1002/uog.22034.
28. Karami P, Naghavi M, Feyzi A, Aghamohammadi M, Novin MS, Mobaien A, et al. Mortality of a pregnant patient diagnosed with COVID-19: a case report with clinical, radiological, and histopathological findings. Travel Med Infect Dis. 2020;doi: 10.1016/j.tmaid.2020.101665 [Epub ahead of print].
29. Liao Z, Yang H, Kong J, Yang H. Chest CT findings in a pregnant patient with 2019 Novel Coronavirus Disease. Balkan Med J. 2020;37(4):226-8.
30. Li M, Xu M, Zhan W, Han T, Zhang G, Lu Y. Report of the first cases of mother and infant infections with 2019 novel coronavirus in Xinyang City Henan Province. Chin J Infect Dis. 2020;38(0):E007.
31. Yao L. Asymptomatic COVID-19 infection in pregnant woman in the third trimester: a case report. Chin J Perinatal Med. 2020;23(3):229-31.
32. Zhuang S, Guo J, Cao Y, Chen H, Xu D, Li J, et al. Perinatal novel coronavirus infection: a case report. Chin J Perinatal Med. 2020;23(2):85-90.
33. Wen R, Sun Y, Xing Q. A patient with SARS-CoV-2 infection during pregnancy in Qingdao, China. J Microbiol Immunol Infec. 2020;53(3):499-500.
34. Zambrano LI, Fuentes-Barahona IC, Bejarano-Torres DA, Sanchez-Martinez FE, Valle-Recono JA, Sierra M, et al. A pregnant woman with COVID-19 in Central America. Travel Med Infect Dis. 2020:101639. doi: 10.1016/j.tmaid.2020.. Online ahead of print.
35. Xiong X, Wei H, Zhang Z, Chang J, Ma X, Gao X, et al. Vaginal delivery report of a healthy neonate born to a convalescent mother with COVID19. J Virol. 2020;First published: 10 April 2020 https://doi.org/10.1002/jmv.25857.
36. Alzamaro MC, Paredes T, Caceres D, Webb CM, Valdez LM, Rosa ML. Severe COVID-19 during pregnancy and possible vertical transmission. Am J Perinatol. 2020;37(8):861-65.
37. Peng Z, Wang J, Mo Y, Duan W, Xiang G, Yi M, et al. Unlikley SARS-CoV-2 vertical transmission from mother to child: a case report. J Infect Pub Health. 2020;13(5):818-20.
38. Lee DH, Lee J, Kim E, Woo K, Park HY. Emergency caesarean section performed in a patient with confirmed severe acute respiratory syndrome Coronavirus-1: a case report. Kor J Anetheriol. 2020;DOI: https://doi.org/10.4097/kja.20116.
39. Li Y, Zhao R, Zheng S, Chen X, Wang J, Sheng X, et al. Lack of vertical transmission of severe acute respiratory syndrome Coronavirus 2, China. Emerg Infect Dis. 2020;26(6):1335-36.
40. Wang S, Guo L, Chen L, Liu W, Cao Y, Zhang J, et al. A case report of neonatal 2019 Coronavirus Disease in China. Clin Infect Dis. 2020;71(15):853-57.
41. Wang X, Zhou Z, Zhang J, Zhu F, Tang Y, Shen X. A case of 2019 Novel Coronavirus in a pregnant woman with preterm delivery. Clin Infect Dis. 2020;71(15):844-46.
42. Gidlof S, Savhenko J, Brune T, Josefsson H. COVID-19 in pregnancy with comorbidities: more liberal testing strategy is needed. Acta Obstetricia et Gynecologica Scndinavia. 2020;First published: 06 April 2020 https://doi.org/10.1111/aogs.13862.
43. Iqbal S, Overcash R, Mokhtari N, Saeed H, Gold S, Auguste T, et al. An uncomplicated delivery in a patient with Covid-19 in the United States. N Eng J Med. 2020;382:e34.
44. AC. M, Sagedal LR, Krogedal TS, Mcfadzean I, Oommen H. High risk delivery patient with COVID-19. Tidsskr Not Legeforen. 2020;DOI: 10.4045/tidsskr.20.0425.

45. Makwe CC, Okunade KS, Rotimi MK, Ekor OE, Oyeleke OG, Bello QO, et al. Cesarean delivery of first prediagnosed COVID-19 pregnancy in Nigeria PanAfrican Medical Journal. 2020;36:100.
46. Kinsey KE, Ganz E, Khalil S, Brustman L. Intraoperative coagulopathy during cesarean section as an unsuspected intial presentation of COVID-19: a case report. BMC Preg Childbirth. 2020;20:481.
47. Figueiredo R, Falco V, Pinto MJ, Ramalho C. Peripheral facial paralysis as presenting symptom of COVID-19 in a pregnant woman. BMJ. 2020;13:e237146.
48. Naqvi M, Zakowski P, Glucksman L, Smithson S, Burwick RM. Case report: Tocilizumab and Remdesivir in a pregnant patient with coronavirus disease 2019 (COVID-19). Obstet Guecol. 2020:1-5.
49. Peng J, Li R, Yin H, Tang F, Xie H, Li M, et al. A case report of a pregnant woman infected with coronavirus disease 2019 pnuemonia Clinical Case Report. 2020;99:30.
50. Lv Y, Gu B, Chen Y, Hu S, Ruan T, Xu G, et al. No intrauterine vertical transmission in pregnancy with COVID-19: a case report Journal Infect Chemother. 2020;https://doi.org/10.1016/j.jiac.2020.07.015.
51. Wen R, Sun Y, Q X. A Patient with SARS-CoV-2 infection during pregnancy in Qingdao, China. Journal of Microbiology, Immunology and Infection. 2020;https://doi.org/10.1016/j.jmii.2020.03.004.
52. Bae J, Ha J, Kwon M, Park H, Seong W, Hong S. A case of delivery of a pregnant woman with COVID-19 infection in Daegu, Korea. Obstet Gynecol Sci. 2020;63(745-49).
53. Rodriguez AG, Contreras SM, Manovel SMF, Vidal JMM, Buron FD, Fernandez CF, et al. SARS-COV-2 infection during pregnancy, a

risk factor for eclampsia or neurological

manifestations of COVID-19? Case report. BMC Pregnancy and Childbirth. 2020;20:587.
54. Rashidian T, Sharifi N, Fathnezhad-Kazemi A, Mirzamrajani F, Nourollahi S, Ghaysouri A. Death of a neonate with suspected coronavirus disease 2019 born to a mother with coronavirus disease 2019 in Iran: a case report. J of Med Case Reports. 2020;14:186.
55. Gulersen M, Staszewski C, Grayer E, Tam ET, Gottesman E, Isseroff D, et al. Coronavirus Disease 2019 (COVID-19)–Related Multisystem Inflammatory Syndrome in a Pregnant Woman. Obstet Gynecol. 2020;DOI: 10.1097/AOG.0000000000004256.
56. Patel P, Kulkarni S, Guerrero M, Persaud C, Zuberi J, Rebein B. Emergency cesarean section at 38 weeks of gestation with COVID-19 penumonia: a case report Am J Case Rep. 2020;21:e926591.
57. Mark A, Crumley JP, Rudolph KL, Doerschug K, Krupp A. Maintaining Mobility in a Patient Who Is Pregnant and Has COVID-19 Requiring Extracorporeal Membrane Oxygenation: A Case Report. Oxford Uni PRess. 2020.
58. Parsa Y, Shokri N, Jahedbozorgan T, Naeiji Z, Zadehmodares S, Moridi A. Possible Vertical Transmission of COVID-19 to the Newborn; a Case Report. Arch Acad Emerg Med. 2020;9:e5.
59. Kouas S, Zoukar O, Ikridih K, Mahdhi S, Belghaieb I, Haddad A. Infection COVID-19 chez la femme enceinte: à propos d'un cas. PAMJ. 2020;37(156):1-6.
60. Rodrigues ML, Fonseca DF, Gasparinho G, Sepulveda F, Matos T. Signs suggestive of congenital SARS-CoV-2 infection with intrauterine fetal death: A case report. Eur J Obstet Gynecol Reprod Biol. 2021;256:502-26.
61. Michel A, De Logivierea V, Schnuriger A, Lefebvre M, Maisonneuve E, Kayem G. Description of a late miscarriage case at 16 Weeks of Gestation associated with a SARS-CoV-2 infection. J Gyncol Obstet Human Repord. 2021;50:102064.
62. McCarthy CM, Hayes-Ryan D, Harrity C, Hogan J, Roopnarinesingh R, O'Dwyer V. A Rare Coincidence—a Second Trimester Ectopic Pregnancy Following Early Medical Abortion: a Case Report. SN Compr Clin Med. 2021;doi.org/10.1007/s42399-021-00748-z.
63. Rana MS, <. U, Alam MM, Am K, Ikram A, Salman M, et al. First trimester miscarriage in a pregnant woman infected with COVID-19 in Pakistan. J Infect. 2021;82:e27-8.
64. Alwardi TH, Ramdas V, Yahmadi MA, Aisari SA, Bhandari S, Hashami HSA, et al. Is vertical transmission of sars-cov-2 infection possible in preterm triplet pregnancy? a case series. Ped Infect Dis J. 2020;39(12):e456-58.
65. Hirshberg A, Pierce-Williams R, Short WR, Parry S, Berghella V, Treibwasser JE. Care of critically ill pregnant patients with coronavirus disease 2019: a case series Am J Obstet Gynecol. 2020;223(2):286-90.

66. Barbero P, Mugüerza L, Herraiz I, Burguillo AG, San Juan R, Forcén L, et al. SARS-CoV-2 in pregnancy: characteristics and outcomes of hospitalized and non-hospitalized women due to COVID-19. Journal of Maternal-Fetal and Neonatal Medicine. 2020 https://doi.org/10.1080/14767058.2020.1793320.
67. Sutton D, Fuchs K, D'Alton M, Goffman D. Universal screening for SARS-CoV-2 in women admitted for delivery. N Eng J Med. 2020;382(22):2163-64.
68. Mayor S. Covid-19: nine in 10 pregnant women with infection when admitted for delivery are asymptomatic, small study finds. BMJ. 2020;369::m1485.
69. Andrikopoulou M, Madden N, Wen T, Aubey JJ, Aziz A, Baptiste CD, et al. Symptoms and Critical Illness Among Obstetric Patients With Coronavirus Disease 2019 (COVID-19) Infection. Obstet Gynecol. 2020 May 27;doi: 10.1097/AOG.0000000000003996. Online ahead of print.
70. Wang Z, Wang Z, Xiong G. Clinical characteristics and laboratory results of pregnant women with COVID-19 in Wuhan, China. Int J Gynecol Obstet. 2020;382:E100.
71. Li N, Han L, Peng M, Lv Y, Ouyang Y, Liu K, et al. Maternal and neonatal outcomes of pregnant women with COVID-19 pneumonia: a case-control study. Clin Infect Dis. 2020;https://doi.org/10.1101/2020.03.10.20033605.
72. Chen R, Zhang Y, Huang L, Cheng B, Xia Z, Meng Q. Safety and efficacy of different anaesthetic regimens for parturient with COVID-19 undergoing caesarean delivery: a case series of 17 patients. Can J Anesth. 2020;67(6):655-63.
73. Li L, Liu D. Follow-up information about the four pregnant patients with coronavirus disease (COVID-19) pneumonia who were still in the hospital at the end of our study. AJR 2020;215:W1-2.
74. Li L, Liu D, Yang L. Reply to "CT findings of pregnant women with coronavirus disease (COVID-19) pnuemonia. AJR. 2020;215:W1.
75. Liu D, Li L, Wu X, Zheng D, Wang J, Yang L, et al. Pregnancy and perinatal outcomes of women with Coronavirus Disease (COVID-19) Pneumonia: a preliminary analysis. AJR. 2020;215:1-6.
76. Liu H, Liu F, Li J, Zhang T, Wang D, Lan W. Clinical and CT imaging features of the COVID-19 pneumonia: focus on pregnant women and children. J Infect. 2020;https://doi.org/10.1016/j.jinf.2020.03.00.
77. Yang P, Wang X, Liu P, Wei C, He B, Zheng J, et al. Clinical characteristics and risk assessment of newborns born to mothers with COVID-19. J Clin Virol. 2020;127:104356.
78. Breslin N, Baptiste C, Gymafi-Bannerman C, Miller R, Martinez R, Bernstein K, et al. COVID-19 infection among asymptomatic and symptomatic pregnant women: two weeks of confirmed presentation to an affiliated pair of New York City Hospitals. Am J Obstet Gynecol MFM. 2020;2(2):100118. doi: 10.1016/j.ajogmf.2020.. Online ahead of print.
79. Breslin N, Baptiste C, Miller R, Fuchs K, Goffman D, Gyamfi-Bannerman C, et al. COVID-19 in pregnancy: early lessons. Am J Obstet Gynecol. 2020;2(2):100111. doi: 10.1016/j.ajogmf.2020.. Epub 2020 Mar 27.
80. Chen H, Guo J, Wang C, Luo F, Yu X ZW, Li J, et al. Clinical characteristics an intrauterine vertical transmission potential of COVID-19 infection in nine pregnant women: a retrospective review of medical records. Lancet. 2020;395(10226):809-15.
81. Lei D, Wang C, Li C, Fang C, Yang W, Chen B, et al. Clinical characteristics of COVID-19 in pregnancy: analysis of nine cases. Chin J Perinatal Med. 2020;23(3):222-28.
82. Chen S, Liao E, Cao D, Gao Y, Sun G, Shao Y. Clinical analysis of pregnant women with 2019 novel coronavirus pneumonia. J Med Virol. 2020;First published 28 March 2020 https://doi.org/10.1002/jmv.25789.
83. Chen Y, Peng H, Wang L, Zhao Y, Zeng L, Gao H, et al. Infants born to mothers with a new coronavirus (COVID-19). Front Pediatr. 2020;Published online 16 March 2020. https://doi.org/10.3389/fped.2020.00104.
84. Fan C, Lei D, Fang C, Li C, Wang M, Liu Y, et al. Perinatal transmission of COVID-19 associated SARS-COV-2: should we worry? . Clin Infect Dis. 2020;ciaa226, https://doi.org/10.1093/cid/ciaa226.
85. Shuo C, Bo H, Danju L, Xiang L, Fan Y, Yin Z, et al. Pregnant women with new coronavirus infection: a clinical characteristics and placental pathological analysis of three cases. Chinese J Obstet Gynecol. 2020;49(0).
86. Yu N, Li W, Kang Q, Xiong Z, Wang S, Lin X, et al. Clinical features and obstetric and neonatal outcomes of pregnant patients with COVID-19 in Wuhan, China: a retrospective, single-centre, descriptive study. Lancet Infect Dis. 2020;20:559-64.

87. Zhu H, Wang L, Fang C, Peng S, Zhang L, Chang G, et al. Clinical analysis of 10 neonates born to mothers with 2019-nCoV pneumonia. Tansl Pediat. 2020;9(1):51-60.
88. Yang H, Sun G, Tang F, Peng M, Gao Y, Peng J, et al. Clinical features and outcomes of pregnant women suspected of coronavirus disease 2019. J Infect. 2020;81(1):e40-4.
89. Wu C, Yang W, Wu X, Zhang T, Zhao Y, Ren W, et al. Clinical manifestation and laboratory characteristics of SARS-CoV-2 infection in pregnant women. Virologica Sinica. 2020;35(3):305-10.
90. Cao D, Yin H, Chen J, Tang F, Peng M, Li R, et al. Clinical analysis of ten pregnant women with COVID-19 in Wuhan, China: a retrospective study. Int J Infect Dis. 2020;95:294-300.
91. Ferrazzi E, Frigerio L, Savasi V, Vergani P, Prefumo F, Barresi S, et al. Vaginal delivery in SARS-CoV-2 infected pregnant women in northern Italy: a retrospective analysis. BJOG. 2020;First published: 27 April 2020 https://doi.org/10.1111/1471-0528.16278.
92. Wu Y, Liu C, Dong L, Zhang C, Chen Y, Liu J, et al. Coronavirus disease 2019 among pregnant Chinese women: case series data on the safety of viginal birth and breastfeeding BJOG. 2020;First published: 05 May 2020 https://doi.org/10.1111/1471-0528.16276.
93. Govind A, Essien S, Karthikeyan A, Fakokunde A, Janga D, Yoong W, et al. Re: Novel coronavirus COVID-19 in late pregnany: outcomes of first nine cases in an inner city London hospital. Eur J Obstet Gynecol Reprod Biol. 2020;251:272-74.
94. Liu W, Wang J, Li W, Zhou Z, Liu S, Rong Z. Clinical charcateristics of 19 neonates born to mothers with COVID-19 Front Med 2020;https://doi.org/10.1007/s11684-020-0772-y.
95. Yang H, Hu B, Zhan S, Yng L, ZXiong G. Effects of SARS-CoV-2 infection on pregnant women and their infants: A retrospective study in Wuhan, China. Arch Pathol Lab Med 2020;doi: 10.5858/arpa.2020-0232-SA.
96. Cooke WR, CGleeson S, Jacques A, Place K, Siddall J, Walden A, et al. SARS-CoV-2 infection in very preterm pregnancy: Experiences from two cases. Eur J Obstet Gynecol Reprod Biol. 2020;250:259-60.
97. Zeng Y, Lin L, Qin Q, Wei W, Yang BX, Huang R, et al. Update on clinical outcomes of women with COVID-19 during pregnancy. Int J Gynecol Obstet. 2020;First published: 21 May 2020 https://doi.org/10.1002/ijgo.13236.
98. Fox NS, Melka S. COVID-19 in Pregnant Women: Case Series from One Large New York City Obstetrical Practice. Am J Perinatol. 2020;37(10):1002-04.
99. Chen S, Huang B, Luo DJ, Li X, Yang F, Zhao Y, et al. Pregnancy With New Coronavirus Infection: Clinical Characteristics and Placental Pathological Analysis of Three Cases. Zhonghua Bing Li Xue Za Zhi. 2020;49(5):418-23.
100. Vivanti AJ, Mattern J, Vauloup-Fellous C, Jani J, Rigonnot L, Hachem LE, et al. Retrospective descriptive of pregnant women infected with severe acute respiratory syndrome coronavirus 2, France Emerging Infect Disease 2020;26(9):2069-76.
101. Yue L, Han L, Li Q, Zhong M, Wang J, Wan Z, et al. Anaesthesia and infection control in cesarean section of pregnant women with coronavirus disease 2019 (COVID-19). MedRxiv. 2020;doi: https://doi.org/10.1101/2020.03.23.20040394.
102. Hu X, Gao J, Luo X, Feng L, Liu W, Chen J, et al. Severe Acute Respiratory Syndrome Coronavirus 2 (SARS-CoV-2) Vertical Transmission in Neonates Born to Mothers With Coronavirus Disease 2019 (COVID-19) Pneumonia. Obstet Gynecol. 2020;136(65-67).
103. Blitz MJ, Rochelson B, Minkoff H, Meirowitz N, Prasannan L, London V, et al. Maternal mortality among women with coronavirus disease 2019 admitted to the intensive care unit. AJOG. 2020;https://doi.org/10.1016/j.ajog.2020.06.020:1-4.
104. Blitz MJ, Grunbaum A, E B. Intensive care unit admissions for pregnant and nonpregnant women with coronavirus disease 2019. AJOG. 2020:290-91.
105. Zhnag L, Dong L, Ming L, Wei M, Li J, Hu R, et al. Severe acute respiratory syndrome coronavirus 2(SARS-CoV-2) infection during late pregnancy: a report of 18 patients from Wuhan, China. BMC Pregnancy Childbirth. 2020;20:394.
106. Xu S, Shao F, Bao B, Ma X, Xu Z, You J, et al. Clinical Manifestation and Neonatal Outcomes of Pregnant Patients With Coronavirus Disease 2019 Pneumonia in Wuhan, China. Open Forum Infectious Diseases 2020:1-7.
107. Wang S, Wei L, Gao X, Chen S, Zeng W, Wu J, et al. Clinical characteristics and outcomes of childbearingage women with Coronavirus disease 2019 in Wuhan: a retrospective, single-center study. J Med Internet Res. 2020;https://preprints.jmir.org/preprint/19642.

108. Chen Y, Bai J. Maternal and infant outcomes of full-term pregnancy combined with COVID-2019 in Wuhan, China: retrospective case series. Arch Gynecol Obstet. 2020;https://doi.org/10.1007/s00404-020-05573-8.
109. Wu Y, Liu J, Xu J, Chen Y, Yang W, Chen Y, et al. Neonatal outcome in 29 pregnant women with COVID-19: A retrospective study in Wuhan, China. PLoS Med. 2020;17(7):e1003195.
110. Cheng B, Jiang T, Zhang L, Hu R, Tian J, Jiang Y, et al. Clinical Characteristics of Pregnant Women With Coronavirus Disease 2019 in Wuhan, China. Open Forum Infectious Diseases. 2020:1-9.
111. Gabriel MAM, Cuadrado I, Fernandez BA, Carrasco EG, Diaz CA, Martin IL, et al. Multicentre Spanish study found no incidences of viral transmission in infants born to mothers with COVID-19. Acta Pedriatica. 2020:1-7.
112. Antoun L, Taweel NE, Ahmed I, Patni S, Honest H. Maternal COVID-19 infection, clinical characteristics, pregnancy, and neonatal outcome: A prospective cohort study. Eur J Obstet Gynecol Biol. 2020;https://doi.org/10.1016/j.ejogrb.2020.07.008.
113. Perlman J, Oxford C, Chang C, Salvatore C, Pace JD. Delivery Room Preparedness and Early Neonatal Outcomes During COVID-19 Pandemic in New York City. Pediatrics. 2020;146(2):e20201567.
114. Zeng L, Xia S, Yuan W, Yan K, Xiao F, Shao J, et al. Neonatal Early-Onset InfectionWith SARS-CoV-2 in 33 Neonates Born to MothersWith COVID-19 inWuhan, China. JAMA Pediatrics. 2020;174(7):722-25.
115. Ahmed I, Azhar A, Eltaweel N, Tan BK. First Covid-19 maternal mortality in the UK associated with thrombotic complications. Br J Haematol. 2020;190(1):e37-e8.
116. Liu P, Zheng J, Yang P, Wang X, Wei C, Zhang S, et al. The immunologic status of newborns born to SARS-CoV-2–infected mothers in Wuhan, China. J Allergy Clin Immunol. 2020;146:101-9.
117. Zhou R, Chen Y, Lin C, Li H, Cai X, Cai Z, et al. Asymptomatic COVID-19 in pregnant woman with typical chest CT manifestation: a case report. Chin J Perinatal Med. 2020;23(3):e006.
118. Liu W, Cheng H, Wang J, Ding L, Zhou Z, Liu S, et al. Clinical Analysis of Neonates Born to Mothers with or without COVID-19: A Retrospective Analysis of 48 Cases fromTwo Neonatal Intensive Care Units in Hubei Province. Am J Perinatol. 2020;37:1317-23.
119. Sahin D, Tanacan A, Erol SA, Anuk AT, Eyi EGY, Ozgu-Erdinc S, et al. A pandemic centre's experience of managing pregnant women with COVID-10 infection in Turkey " a prospective cohort study Obstet Gynecol. 2020;https://doi.org/10.1002/ijgo.13318.
120. Woodworth KR, Olsen EO, Neelam V, Lewis EL, Galang RR, Oduyebo T, et al. Birth and Infant Outcomes Following Laboratory-Confirmed SARS-CoV-2 Infection in Pregnancy — SET-NET, 16 Jurisdictions, March 29–October 14, 2020. Morbidity and Mortality Weekly Report. 2020;69(44):1635-40.
121. DeBolt CA, Bianco A, Limaye MA, Silverstein J, Penfield CA, Roman AS, et al. Pregnant women with severe or critical COVID-19 have increased composite morbidity compared to non-pregnant matched controls. Am J Obstet Gynecol. 2020;https://doi.org/10.1016/j.ajog.2020.11.022.
122. Yang R, Mei H, Zheng T, Fu Q, Zhang Y, Buka S, et al. Pregnant women with COVID-19 and risk of adverse birth outcomes and maternal-fetal vertical transmission: a population-based cohort study in Wuhan, China. BMC Med. 2020;18:330.
123. Dilahoy MJ, Whitaker M, O'Halloran A, Chai SJ, Kirley PD, Alden N. Characteristics and Maternal and Birth Outcomes of Hospitalized Pregnant Women with Laboratory-Confirmed COVID-19 — COVID-NET, 13 States, March 1–August 22, 2020. MMWR. 2020;69(38):1347-54.
124. Panagiotakopoulos L, Myers TR, Gee J, Lipkind HS, Kharbanda EO, Ryan DS. SARS-CoV-2 Infection Among Hospitalized Pregnant Women: Reasons for Admission and Pregnancy Characteristics — Eight U.S. Health Care Centers, March 1–May 30, 2020. MMWR. 2020;69(38):1355-59.
125. Lumbreas-Marquez MI, Campos-Zamora M, de Leon HL, Farber MK. Maternal mortality from COVID-19 in Mexico. Int J Gynecol Obstet. 2020;First published: 30 May 2020 https://doi.org/10.1002/ijgo.13250.
126. Ellington S, Strid P, Tong VT, Woodworth K, Galang RR, Zamberno LD, et al. Characteristics of women of reproductive age with laboratory-confirmed SARS-CoV-2 infection by pregnacy status - United Staes, January 22-June 7, 2020. Morbidity and Mortality Weekly Report 2020;69:769-75.
127. Ozsurmeli M, Terzi H, Hocaoglu M, Bilir RA, Guay T, Unsal D, et al. Clinical characteristics, maternal and neonatal outcomes of pregnant women with SARS-CoV-2 infection in Turkey. Bratisl Med J. 2021;122(2):152-57.
128. Martinez-Portilla RJ, Sotiriadis A, Chatzakis C, Torres-Torres J, Sosa AEY, Sandoval-Mandujano K, et al. Pregnant women with SARS-CoV-2 infection are at higher risk of death and pneumonia: propensity score

matched analysis of a nationwide prospective cohort (COV19Mx). Ultrasound Obstet Gynecol. 2021;57:224-31.
129. Maru S, Patil U, Carroll-Bennett R, Baum A, Bohn-Hemmerdinger T, Ditchik A, et al. Universal screening for SARS-CoV-2 infection among pregnant women at Elmhurst Hospital Center, Queens, New York. PloS ONE. 2020;15(12):e0238409.
130. Gabriel MAM, Vergeli MR, Carbonero SC, Sole L, Molina TC, Calle IR, et al. Maternal, Perinatal and Neonatal Outcomes With COVID-19: A Multicenter Study of 242 Pregnancies and Their 248 Infant Newborns During Their First Month of Life. Ped Infect Dis J. 2020;39(12):e393-97.
131. Cruz-Lemini M, Perez EF, Conty MLC, Aguilar AC, Pardilla MBE, Rodriguez PP, et al. Obstetric Outcomes of SARS-CoV-2 Infection in Asymptomatic Pregnant Women. Viruses. 2021;13:112.
132. Guo Y, Yuan J, Wang M, Yu Y, Bian J, Fan C. Case series of 20 pregnant women with 2019 novel coronavirus disease in Wuhan, China. J Obstet Gyncol Res. 2021;doi:10.1111/jog.14664 J.
133. Ochiai D, Kasuga Y, Lida M, Ikenoue S, Tanaka M. Universal screening for SARS-CoV-2 in asymptomatic obstetric patients in Tokyo, Japan. Int J Gynecol Obstet. 2020;First published: 04 June 2020 https://doi.org/10.1002/ijgo.13252.
134. Chang W. COVID-19 pandemic's effects on the quality of pregnant women's emergency treatment: Review of two cases from a medical center in northern Taiwan. Taiwanese J Obstet & Gyncecol. 2020;59:795-800.
135. Tang J, Song W, Xu H, Wang N. No evidence for vertical transmission of SARSCoV-2 in two neonates with mothers infected in the second trimester. Infect Dis. 2020;52(12):913-16.
136. Cao D, Chen M, Peng M, Yin H, G S. Vaginal delivery in women with COVID-19: report of two cases. BMC Pregnancy and Childbirth. 2020;20:580.
137. Gheysarzadeh A, Sadeghifard N, Safari M, Rashidian T, Mohammadyari E, Tavan H. Case series of four pregnant women with COVID-19 in Ilam, Iran. New Microme New Infect. 2020;38:100783.
138. dos Reis HLB, Boldrini NAT, Caldas JVJ, da Paz APC, Ferrugini CLP, Miranda AE. Severe coronavirus infection in pregnancy: challenging cases report. Journal of the Sao Paulo Insstitute of Tropical Medicine 2020:1-5.
139. Buonsenso D, Costa S, Sanguinetti M, Cattani P, Posteraro B, Marchetti S, et al. Neonatal late onset infection with severe acute respiratory syndrome Coronavirus 2. Am J Perinatol. 2020;37(8):869-72.
140. Perrone S, Deolmi M, Giordano M, D'Alvano T, Gambini L, Corradi M, et al. Report of a Series of Healthy Term Newborns From Convalescent Mothers With COVID-19. Acta Biomed. 2020;91(2):251-55.
141. Gagliardi L, Danieli R, Suriano G, Vaccaro A, Tripodi G, Rusconi F, et al. Universal severe acute respiratory syndrome coronavirus 2 testing of pregnant women admitted for delivery in 2 Italian regions American Journal of Obstetrics and Gynecology. 2020:291-92.
142. Romagano MP, Guerreo K, Spillane N, Kayaalp E, Smilen SW, Alvarez M, et al. Perinatal outcomes in critically ill pregnant women with coronavirus disease 2019. AJOG MFM. 2020:1-7.
143. Juusela A, Nazir M, Gimovsky M. Two cases of COVID-19 related cardiomyopathy in pregnancy. Am J Obstet Gynecol MFM. 2020;2(2):100113.
144. Lucarelli E, Behn C, Lashley S, Smok D, Benito C, POyelese Y. Mechanical Ventilation in Pregnancy Due to COVID-19: A Cohort of Three Cases. Am J Perinatol. 2020;37(10):1066-69.
145. Sun M, Xu G, Yang Y, Tao Y, Pian-Smith M, Mashavan V, et al. Evidence of mother-to-newborn infection with COVID-19. Br J Anesthesia. 2020;125(2):e245-57.
146. Khan S, Peng L, Siddique R, Nabi G, Nawsherwan MS, Xue M, et al. Impact of CVID-19 infection on pregnancy outcomes and the risk of maternal-to-neonatal intrapartum transmission of COVID-19 during natural birth. Infect Control Hosp Epidemiol. 2020;doi: 10.1017/ice.2020.84:1-3.
147. Chen X, Li Y, Wang J, Cai H, Cao H, Sheng J. Pregnant women complicated with COVID-19: a clinical analysis of 3 cases Zhejiang Da Xue Xue Bao Yi Xue Ban. 2020;49(2):240-9.
148. Wenhui W, Zhao Z, He Z, Liu S, Wu Q, Zhang X, et al. Unfavorable outcomes in pregnant patients with COVID-19. J Infect. 2020;81(2):e99-101.
149. Zheng T, Guo J, He W, Wang H, Yu H, Ye H. Coronavirus disease 2019 (COVID-19) in pregnancy: 2 case reports on maternal and neonatal outcomes in Yichang city, Hubei Province, China. Clinical Case Report. 2020;http://dx.doi.org/10.1097/MD.0000000000021334:1-7.
150. Hantoushzadeh S, Shamshirasaz AA, Aleyasin A, Seferovic MD, Aski SK, Arian SE, et al. Maternal death due to COVID-19. Am J Obstet Gynecol. 2020;223(1):109e1-e16.

151. Olivini N, Carducci FIC, Santilli V, De Ioris MA, Scarselli A, Alario D, et al. A neonatal cluster of novel coronavirus disease 2019: clinical management and 1considerations. Italian J Pediatr. 2020;46:180.
152. Fashner J, Cintron C. Nine SARS-CoV-2 Positive Pregnant Women and Their Infant Delivery Outcomes. Cureus. 2020;12(12):e11946.
153. Chen Y, Li Z, Zhang Y, Zhao W, Yu Z. Maternal health care management during the outbreak of coronavirus disease 2019 (COVID-19). J Med Virol. 2020;First published: 26 March 2020 https://doi.org/10.1002/jmv.25787.
154. Tassis B, Lunghi G, Frattaruolo, Ruggiero M, Somigliana E, Ferrazzi E. Effectiveness of a COVID-19 screening questionnaire for pregnant women at admission to an obstetric unit in Milan. Int J Obstet Gynecol. 2020;DOI: 10.1002/ijgo.13191.
155. Khalil A, Hill R, Ladhani S, Pattisson K, O'Brien P. Severe acute respiratory syndrome coronavirus 2 in pregnancy: symptomatic pregnant women are only the tip of the iceberg. AJOG. 2020;https://doi.org/10.1016/j.ajog.2020.05.005:296-7.
156. Molteni E, Astley CM, Ma W, Sudre CH, Magee LA, Murray B, et al. SARS-CoV-2 (COVID-19) infection in pregnant 1 women: characterization of symptoms and syndromes predictive of disease and severity through real-time, remote participatory epidemiology. MedRxiv. 2020;https://doi.org/10.1101/2020.08.17.20161760.
157. Freiesleben NlC, Egerup P, VHviid KVR, Severinsen ER, Kolte AM, Westergaard D, et al. SARS-CoV-2 in first trimester pregnancy – does it affect the fetus? medRxiv. 2020;https://doi.org/10.1101/2020.06.08.20125195.
158. Cosma S, Carosso A, Cusato J, Borella F, Carosso M, Bovetti M, et al. COVID-19 and first trimester spontaneous abortion: a case-control study 1 of 225 pregnant patients. Am J Obstet Gynecol. 2020;https://doi.org/10.1016/j.ajog.2020.10.005:e1-e7.
159. Liu W, Wang Q, Zhang Q, Chen L, Chen J, Zhang B, et al. Coronavirus disease 2019 (COVID-19) during pregnancy: a case series. Preprints. 2020;preprints.org > 202002.0373.v1.
160. Dong Y, Mo X, Hu Y, Tong S. Epidemiological and transmission patterns of pregnant women with 2019 coronavirus disease in China. SSRN. 2020; https://ssrn.com/abstract=3551330 or http://dx.doi.org/10.2139/ssrn.3551330.
161. Massorotti C, Adriana M, Cagnacci A, Gorlero F, Gustavino C, Vallerino G, et al. Asymptomatic SARS-CoV-2 infections in pregnant patients in an Italian city during complete lockdown. J Med Virol. 2020;https://doi.org/10.1002/jmv.26458.
162. Waghmare R, Gajbhiye R, Mahajan NN, Modi D, Mukherjee S, Mahale SD. Universal screening identifies asymptomatic carriers of SARS-CoV-2 among pregnant women in India. Eur J Obstet Gynecol Reprod Biol. 2021;256:502-26.
163. Saccone G, Sen C, Mascio DD, Galindo A, Grunebaum A, Yoshimatsu J, et al. Maternal and Perinatal Outcomes of Pregnant Women with Coronavirus Disease 2019 (COVID-19): The WAPM study on COVID-19. Lancet Infectious Diseases 2020.
164. Mascio DD, Sen C, Saccone G, Galindo A, Grünebaum A, Yoshimatsu J, et al. Risk factors associated with adverse fetal outcomes in pregnancies affected by Coronavirus disease 2019 (COVID-19): a secondary analysis of the WAPM study on COVID-19. J Perinatol Med. 2020;48(9):950-58.
165. Villar J, Ariff S, Gunier RB, Thiruvengadam R, Rauch S, Kholin A, et al. Maternal and neonatal morbidity and mortality among pregnant women with and without COVID-19 infection: the INTERCOVID multinational cohort study. JAMA Pediatrics 2021. Published online April 22, 2021. doi:10.1001/jamapediatrics.2021.1050
166. Knight M, Bunch K, Vousden N, Morris E, Simpson N, Gale C, et al. Characteristics and outcomes of pregnant women admitted to hospital with confirmed SARS-CoV-2 infection in UK: national population based cohort study. BMJ. 2020;369:m2107.
167. Kmietowicz Z. Covid-19: Half of pregnant women in UK hospitals are from ethnic minorities. BMJ. 2020;369:m2266.
168. Doria M, Perixinho C, Laranjo M, Verejao AM, Silva PT. Covid-19 during pregnancy: A case series from an universally tested population from the north of Portugal. Eur J Obstet Gynecol Biol. 2020;250:261-62.
169. Takemoto MLS, de Menezes MO, Andreucci CB, Nakamura-Pereira M, Amorm MMR, Katz L, et al. The tragedy of COVID-19 in Brazil: 124 maternal deaths and counting Int J Obstet Gynecol. 2020:154-6.
170. NethOSS. Update registration COVID-19 positive pregnant women in 159. 2020.

171. Crovetto F, Crispi F, Llurba E, Figueras F, Gomez-Roig MD, Gratacos E. Seroprevalence and clinical spectrum of SARS-CoV-2 infection in the first versus third trimester of pregnancy MedRxiv. 2020;https://doi.org/10.1101/2020.06.17.20134098.
172. Mendoza M, Garcia -Ruiz I, Maiz N, Rodo C, Garcia-Manau P, Serrano B, et al. Preeclampsia like syndrome induced by severe COVID-19: a prospective observational study. BJOG. 2020;First published: 01 June 2020 https://doi.org/10.1111/1471-0528.16339.
173. San-Juan R, Barbero P, Fernandex-Ruiz M, Lopez-Medrano F, Lizasoain M, Hernandez-Jimenez P, et al. Incidence and clinical profiles of COVID-19 pneumonia in pregnant womne: a single-centre cohort study from Spain. EClinalMedicine. 2020;23:100407.
174. Pereira A, Cruz-Melguizo S, Adrien M, Fuentes L, Marin E, Perez-Medina T. Clinical course of coronavirus disease-2019 (COVID-19) in pregnancy. Am J Obstet Gynecol. 2020;First published: 22 May 2020 https://doi.org/10.1111/aogs.13921.
175. Martínez-Perez O, Vouga M, CruzMelguizo S, Acebal LF, Panchaud A, Chápuli M, et al. Association Between Mode of Delivery Among PregnantWomenWith COVID-19 and Maternal and Neonatal Outcomes in Spain. JAMA. 2020;324(3):296-99.
176. Cohen J, Vignaux O, Jacquemard F. Covid-19 in pregnant women: General data from a French National Survey. Eur J Obstet Gynecol Reprod Biol. 2020;251:267-68.
177. Kayem G, Alessandrini V, Azria E, Blanc J, Bohec C, Bornes M, et al. A snapshot of the Covid-19 pandemic among pregnant women in France. J Gynecol Obstet Human Reprod. 2020;S2468-7847(20)30170-7.
178. Savasi Vm, Parisi F, Patane L, Ferrazzi E, Frigerio L, A. P, et al. Clinical findings and disease severity iin hospitalised pregnant women with coronavirus disease 2019 (Covid-19). Obstet Gynecol. 2020;00:1-7.
179. Maraschini A, Corsi E, Salvatore MA, Donati S, Group IC-W. Coronavirus and birth in Italy: results of a national population-based cohort study. MedRxiv. 2020;https://doi.org/10.1101/2020.06.11.20128652.
180. Pierce-Williams RAM, Burd J, Felder L, Khouray R, PS. B, Avila K, et al. Clinical course of severe and critical COVID-19 in hospitalized pregnancies: a US cohort study. Am J Obstet Gynecol MFM. 2020;doi: https://doi.org/10.1016/j.ajogmf.2020.100134.
181. Guardo FD, DGrazia FMD, Gregorio LMD, Zambrotta E, Carrara G, Gulino FA, et al. Poor maternal–neonatal outcomes in pregnant patients with confirmed SARS-Cov-2 infection: analysis of 145 cases. Arch Gynecol Obstet. 2021;https://doi.org/10.1007/s00404-020-05909-4.
182. Molina EO, Pailos RH, Guillen MP, Pedreno AP, Rodriguez ER, Martinez AH. COVID-19 infection in symptomatic pregnant women at the midpoint of the pandemic in Spain: a retrospective analysis. Ginekologia Polska. 2020;91(12):755-63.
183. Adhikari EH, Moreno W, Zofkie AC, MacDonald L, Mclntrie DD, Collins RRJ, et al. Pregnancy Outcomes AmongWomen With and Without Severe Acute Respiratory Syndrome Coronavirus 2 Infection. JAMA Open. 2020;3(11):e2029256.
184. Hcini N, Maamri F, Picone O, Carod J, Lambert V, Mathieu M, et al. Maternal, fetal and neonatal outcomes of large series of SARS-CoV-2 positive pregnancies in peripartum period: A single-center prospective comparative study. Eur J Obstet Gynecol Reprod Biol. 2021;257(11-18).
185. Campbell KH, Tornatore JM, Illuzzi JL, Sussman LS, Lipkind HS, Pettker CM. Prevalence of SARS-CoV-2 Among Patients Admitted for Childbirth in Southern Connecticut. JAMA. 2020;323:2520-22.
186. Vintzileos WS, Muscat J, Hoffman E, John NS, Vertichio R, Vintzileos AM, et al. Screening all pregnant women admitted to labor and delivery for the virus responsible for coronavirus disease 2019. American Journal of Obstetrics and Gynecology. 2020;https://doi.org/10.1016/j.ajog.2020.04.024:284-86.
187. Prabhu M, Cagino K, Mattews KC, Friedlander RL, Glynn SM, Kubiak JM, et al. Pregnancy and postpartum outcomes in a University tested population for SARS-CoV-2 in New York City: a proscpective cohort study. Obstet Gynecol. 2020; https://doi.org/10.1111/1471-0528.16403.
188. McLaren RA, London V, Ataullah F, McCalla S, Haberman S, Fisher N, et al. Delivery For Respiratory Compromise Among Pregnant Women With COVID-19. Am J Obstet Gynecol. 2020;https://doi.org/10.1016/j.ajog.2020.05.035.
189. Khoury R, Berstein PS, BDebolt C, Stone J, Sutton DM, Simpson LL, et al. Characteristics and Outcomes of 241 Births to Women With Severe Acute Respiratory Syndrome Coronavirus 2 (SARS-CoV-2) Infection at Five New York City Medical Centers. Obstet Gynecol. 2020;136:273-82.

190. London V, McLaren R, Atallah F, Cepeda C, McCalla S, Fisher N, et al. The Relationship between Status at Presentation and Outcomes among Pregnant Women with COVID-19. Am J Perinatol. 2020;37(10):991-94.
191. Lokken EM, Walker CL, Delaney S, Kachikis A, Kretzer NM, Erickson A, et al. Clinical Characteristics of 46 Pregnant Women with a SARS-CoV-2 Infection in Washington State. Am J Obstet Gynecol. 2020;doi: https://doi.org/10.1016/j.ajog.2020.05.031.
192. Duffy CR, Hart JM, Modest AM, Hacker MR, Golen T, Li Y, et al. Lymphopenia and severe acute respiratory syndrome coronavirus 2 (SARS-CoV-2) infection among hospitalized obstetric patients. obstet Gynecol. 2020;136(2):229-31.
193. Goldfarb IT, Clapp MA, Soffer MD, Shook LL, Rushfirth K, Edlow AG, et al. Prevalence and Severity of Coronavirus Disease 2019 (COVID-19) Illness in Symptomatic Pregnant and Postpartum Women Stratified by Hispanic Ethnicity. Obstet Gynecol. 2020;136(2):300-02.
194. Qadri F, Mariona F. PRegnancy affected by SARS-CoV-2 infection: a flash report from Michigin. Journal of Maternal-Fetal and Neonatal Medicine 2020;https://doi.org/10.1080/14767058.2020.1765334:1-3.
195. Griffin I, Benarba F, Peters C, Oyelese Y, Murphy T, Contreras D, et al. The impact of COVID-19 infection on labor and delivery, newborn nursey, and neonatal intesive care unit: propective observational datafrom a single hospital system. Am J Perinatol. 2020;37(10):1022-30.
196. Sakowicz A, Ayala AE, Ukeje CC, Witting CS, Grobman WA, Miller ES. Risk factors for SARS-CoV-2 infection in pregnant women. AJOG MFM. 2020;https://doi.org/10.1016/j.ajogmf.2020.100198.
197. Sahin D, Tanacan A, Erol SA, Anuk AT, Yetiskin FDY, Keskin HL. Updated experience of a tertiary pandemic center on 533 pregnant women with COVID-19 infection: A prospective cohort study from Turkey. Int J Gynecol Obstet. 2020;https://doi.org/10.1002/ijgo.13460.
198. Oncel MY, Akin IM, Kanburoglu MK, Tayman C, Coskun S, Narter F, et al. A multicenter study on epidemiological and clinical characteristics of 125 newborns born to women infected with COVID-19 by Turkish Neonatal Society. European Journal of Pediatrics 2020;https://doi.org/10.1007/s00431-020-03767-5.
199. Yan J, Guo J, Fan C, Juan J, Yu X, Li J, et al. Coronavirus disease 2019 (COVID-19) in pregnant women: A report based on 116 cases. Am J Obstet Gynecol. 2020;DOI:https://doi.org/10.1016/j.ajog.2020.04.014.
200. Lu Z, Yan J, Min W, Biheng C, Xiaocui Z, Jun L, et al. Analysis of the pregnancy outcomes in pregnant women with COVID-19 in Hubei Province. Chinese J of Obstet Gynecol. 2020;55(3):166-71.
201. Chen L, Li Q, Zheng D, Jiang H, Wei Y, Zou L, et al. Clinical characteristic of pregnant women with covid-19 in Wuhan, China. N Eng J Med 2020;382:e100.
202. Liu Y, Chen H, Tang K, Guo Y. Clinical manifestations and outcome of SARS-CoV-2 infection during pregnancy. J Infect. 2020;PII S0163-4453(20)30109-2.
203. KLio J, He X, Gong Q, Yang L, Zhou C, Li J. Analysis of vaginal delivery outcomes among pregnant women in Wuhan, China during the COVID-19 pandemic. Int J Gynecol Obstet. 2020;150:53-7.
204. Qiancheng X, Jian S, Lingling P, Lei H, Xiaogan J, Weihua L, et al. Coronavirus disease 2019 in pregnancy. Int J Obstet Gynecol. 2020;95:376-83.
205. Liu F, Liu H, Hou L, Li J, Zheng H, Chi R, et al. Clinico-radiological features and outcomes in pregnant women with COVID-19 pnuemonia compared with age-matched non-pregnant women. Infection and Drug Resistance. 2020;13:2845-54.
206. Nie R, Wang S, Yang Q, Fan C, Liu Y, He W, et al. Clinical features and the maternal and neonatal outcomes of pregnant women with coronavirus disease 2019. MedRxiv. 2020;https://doi.org/10.1101/2020.03.22.20041061.
207. Mohr-Sasson A, Chayo J, Bart Y, Meyer R, Sivan E, Mazaki-Tovi S, et al. Laboratory characteristics of pregnant compared to non-pregnant women infected with SARS-CoV-2. Archives of Gynecol Obstet.https://doi.org/10.1007/s00404-020-05655-7.
208. Nayak AH, Kapote DS, Fonesca M, Chavan N, Mayekar R, Sarmalkar M, et al. Impact of the coronavirus infection in pregnancy: a preliminary study of 141 pateints J Obstet Gyncol India. 2020;70:256-61.
209. Ceulemans D, Thijs I, Schrers A, Vercammen J, Lannoo L, Deprest J, et al. Screening for COVID-19 at childbirth: is it effective? Ultrasound Obstet Gynecol. 2020;56:110-6.
210. Santhosh J, Salmani MA, Khamis F, Al-Ubaidani SA, Al-Zakwani I. Clinical characteristics of COVID-19 in pregnant women: A retrospective descriptive single-center study from a tertiary hospital in Muscat, Oman. Int J Gynecol Obstet. 2020;00:1-5.

211. Khan MA, Kumar V, SR A. Vertical Transmission of Novel Coronavirus (COVID-19) from Mother to Newborn: Experience from a Maternity Unit, The Indus Hospital, Karachi. J Coll Phy Surg Pak. 2020;30:S136.
212. Mercedes BR, Serwat A, Naffaa L, Ramirez N, Khalid F, Steward SB, et al. New-onset myocardial injury in pregnant patients with coronavirus disease 2019: a case series of 15 patients. Am J Obstet Gynecol. 2020;https://doi.org/10.1016/j.ajog.2020.10.031:e1-e9.
213. Sattari M, Bashirian S, Masoumi SZ, Shayan A, Jenabi E, Ghelichkhani S, et al. Evaluating Clinical Course and Risk Factors of Infection and Demographic Characteristics of Pregnant Women with COVID-19 in Hamadan Province, West of Iran. J Res Health Sci. 2020;20(3):e00488.
214. Jenabi E, Bashirian S, Khazaei S, Masoumi SZ, Ghelichkhani S, Goodarzi F, et al. Pregnancy outcomes among symptomatic and asymptomatic women infected with COVID-19 inthe west of Iran: a case-control study. J Mater Fetal Neonat Med. 2020;https://doi.org/10.1080/14767058.2020.1861599.
215. Aski SK, Sharami SH, Hosseinzadeh F, Hesni E, Heirati SFD, Ghalandari M, et al. Risk Factors, Clinical Symptoms, Laboratory Findings and Imaging of Pregnant Women Infected with COVID-19 in North of Iran. Arch Iran Med. 2020;23(12):856-63.
216. Mattar CNZ, Kalimuddin S, Sadarangani SP, Tagore S, Thain S, Thoon KC, et al. Pregnancy Outcomes in COVID-19: A Prospective Cohort Study in Singapore. Ann Acad Med Singap. 2020;49:857-69.
217. Remaeus K, Savchenko J, Wendel SB, Gidlof S, Wallstrom T. Characteristics and short-term obstetric outcomes in a case series of 67 women tested positive for SARS-CoV-2 in Stockholm, Sweden. Acta Obstet Gynecol Scand. 2020;https://doi.org/10.1111/aogs.14006.
218. Rottenstreich A, Tsur A, Braverman N, Kabiri D, Porat S, Benenson S, et al. Vaginal delivery in SARS-CoV-2-infected pregnant women in Israel: a multicenter prospective analysis. Arch Gynecol Obstet. 2020;https://doi.org/10.1007/s00404-020-05854-2.
219. Farghaly MAA, Kupferman F, Castillo F, Kim RM. Characteristics of Newborns Born to SARS-CoV-2-Positive Mothers: A Retrospective Cohort Study. Am J Perinatol. 2020;37:1310-16.
220. Zambrana LD, Ellington S, Strid P, Galang RR, Oduyebo T, Tong VT. Update: Characteristics of Symptomatic Women of Reproductive Age with Laboratory-Confirmed SARS-CoV-2 Infection by Pregnancy Status — United States, January 22–October 3, 2020. MMWR. 2020;69(44):1641-47.
221. Freiesleben NLC, Egerup P, Hviid KVR, Severinsen ER, Kolte AM, Westergaard D, et al. SARS-CoV-2 in first trimester pregnancy: a cohort study. Eur Soc Human Reprod Embryol. 2020;https://academic.oup.com/humrep.
222. Riod-Silva M, Murillo-Zamora E, Mendoza-Cano O, Trujillo X, Huerta M. COVID-19 mortality among pregnant women in Mexico: A retrospective cohort study. J Glob Health. 2020;10(2):020512.
223. Shmakov RG, Prikhodko A, Polushkina E, Shmakova E, Pyregov A, Bychenko V, et al. Clinical course of novel COVID-19 infection in pregnant women. J Matern Fetal Neonat Med. 2020;https://doi.org/10.1080/14767058.2020.1850683.
224. Ayed A, Embareeg A, Benawadh A, Al-Fouzan W, Hammoud M, Al-Hathal M, et al. Maternal and perinatal characteristics and outcomes of pregnancies complicated with COVID-19 in Kuwait. BMC Pregnancy and Childbirth. 2020;20:754.
